# Transcriptomic effects of alginate hydrogel applied to the production of bovine embryos

**DOI:** 10.1016/j.heliyon.2024.e40957

**Published:** 2024-12-06

**Authors:** Giuliana de A. Ferronato, Paola M. da S. Rosa, Alessandra Bridi, Angélica Camargo dos Santos, Ricardo P. Nociti, Marcos Roberto Chiaratti, Felipe Perecin, Flávio V. Meirelles, Juliano R. Sangalli, Juliano C. da Silveira

**Affiliations:** a Faculty of Animal Science and Food Engineering, Department of Veterinary Medicine, University of São Paulo, Pirassununga, SP, Brazil; b Universidade Federal de São Carlos, Centro de Ciências Biológicas e da Saúde, Departamento de Genética e Evolução, São Carlos, SP, Brazil

## Abstract

*In vitro*-produced blastocysts are exposed to different stimuli when compared with *in vivo* ones. This includes the culture of *in vitro* embryos in a sturdy petri-dish, while *in vivo* embryos develop in a soft and dynamic structure. Here we hypothesized that a softer environment could differently modulate the *in vitro* produced embryos*.* To that aim, presumptive zygotes were produced by *in vitro* fertilization and divided into three groups: 1) Cultured in a regular Petri dish - Control (CON); 2) Cultured on top of an alginate hydrogel surface (TOP); 3) Encapsulated inside an alginate hydrogel sphere (ENC) and cultured. We observed a decrease in blastocyst rate in TOP and ENC compared with the CON. Profiling of 383 bovine miRNAs, we found 3 miRNAs involved in cell proliferation being differently modulated by the TOP and ENC groups (miR-1246; miR-1260b, and miR-541). Analyzing global levels of DNA methylation and hydroxymethylation, we observed increased levels of the two marks in the TOP group when compared with the CON and ENC systems. RNA sequencing (RNA-seq) analysis carried out using blastocysts showed alterations in several developmentally important genes among the three groups. In summary, our results indicate that *in vitro* embryo production was possible to achieve up to the blastocyst stage. However, with the experimental conditions used herein, the alginate hydrogels adversely affected the embryo development, which were paralleled by epigenetic and transcriptomic changes.

## Highlights

• Alginate hydrogel negatively affects embryo culture.
• Embryos cultured in alginate hydrogel have altered DNA global methylation and hydroxymethylation levels.
• Embryos cultured in alginate hydrogel presented alteration in transcriptome.

## 1 Introduction

*In vitro* embryo production (IVP) systems generate blastocysts which differ morphologically and molecularly from blastocysts produced *in vivo* [1]. Blastocysts produced *in vitro* have lower pregnancy rates and resistance to cryopreservation when compared with *in vivo* [2]. In addition, they also have a different pattern of epigenetic marks (DNA methylation, post-translational modifications of histones) and microRNAs (miRNAs) signature caused by the IVP systems [3,4]. These alterations can affect embryo development and newborn health after birth [5]. However, in humans the number of IVP newborns is steadily growing in the world, with more than 8 million babies born thus far, and the number is expected to increase in the next few years [6]. Thus, it is important to look for new IVP approaches aiming to develop more physiological environments.

The early embryonic development is marked by intense chromatin remodeling and Epigenetic reprogramming, making this period particularly sensitive to any type of stress (e.g., culture media, oxygen tension, temperature, type of culture plate) [7]. While *in vivo*, embryos develop in a complex environment inside the oviduct, *in vitro* usually they are cultured in Petri dish plates. Plates are generally composed of polystyrene, with the embryos free in the culture medium, without physical stimuli, that *in vivo* are caused by contact with the maternal tissues [8]. It has been demonstrated that embryos and somatic cells are capable of responding to mechanical stimuli and transforming them into biological signals within the cytoplasm, inducing activation of mechanosensitive pathways that can cause changes in gene transcription within the nucleus [9].

External physical stimuli are important because they can control cell proliferation, differentiation, and growth [10]. Petri or culture dishes are six orders of magnitude (∼10^6^) more rigidity than the uterine epithelium and oviduct [11]. Based on that, softer systems mimicking the *in vivo* environment could improve *in vitro* embryo production, besides contributing to a better understanding of how the early embryo is affected by current IVP conventional systems [12]. An alternative can be the use of hydrogels, which are structures generated from water-soluble polymers, capable of forming three-dimensional networks [13]. There are a large number of polymers that form hydrogels, and the most used in 3D cultures is the alginate [14]. Alginate is a natural polymer obtained from different species of brown algae, which by the addition of a divalent ion, such as calcium, can form a stable hydrogel similar to the extracellular matrix present *in vivo* [15]. Moreover, alginate hydrogels have a rigidity similar to that found in reproductive tissues [11] and it has been shown that alginate does not present cytotoxicity [16]. In bovine, a new approach to bioprint 3D alginate hydrogels was used for COC maturation and was able to improve oocyte bioenergetic/oxidative status, *in vitro* maturation rates as well as transcript levels associated with oocyte competence [17]. Additionally, the alginate hydrogels propitiated a platform to study the process of embryo elongation, which is normally not achieved *in vitro* when considering regular IVP systems [18]. Thus, suggesting it can also be used in other stages of embryo development.

Due to the importance and large use of *in vitro* embryo production in humans and domestic animals, it is critical to explore novel IVP techniques that try to create more physiological conditions. With this in mind, we hypothesize that a softer environment would improve *in vitro* embryo development. To this end, we used alginate polymer as a hydrogel, and the objectives of this study were: 1- To evaluate the development of *in vitro* produced blastocysts in two different culture systems, on top of an alginate surface (TOP) or encapsulated inside an alginate sphere (ENC); 2- To investigate whether the alginate systems affect epigenetic marks in the resulting embryos; 3- To analyze the expression of candidate miRNAs in blastocysts; and 4- To determine the differences in gene expression caused by the different culture environments. Our findings demonstrate that miRNAs involved in cell proliferation were differently modulated by either TOP or ENC treatments. We also observed increased levels of global DNA methylation and hydroxymethylation in the TOP group when compared with the CON and ENC systems. Additionally, RNA sequencing (RNA-seq) analysis identified alteration in several developmentally important genes among the groups. Altogether, these results demonstrated that although the production of blastocysts was possible (albeit smaller compared with control conditions), the alginate hydrogel, in the experimental conditions used herein, negatively affected the bovine embryo development, supported by changes in global levels of epigenetic marks in embryos cultured on alginate layer, and it caused alterations in the transcriptome of the resulting blastocysts cultured on alginate hydrogel layer or encapsulated by alginate hydrogel.

## 2 Materials and methods

### 2.1 Oocyte collection and *in vitro* maturation

In the present work we collected cumulus-oocyte complexes (COCs) from ovaries discarded by a local slaughterhouse. It is worth to note that all experiments conducted in this study were approved by the University of São Paulo Research Ethics Committee (protocol number: 5343150721). The COCs were aspirated from small antral follicles (3–6 mm) and selected in wash media (TCM 199 with Hepes, supplemented with 0.001 g/mL of BSA, 0.2 mM of pyruvate, and 50 μg/mL of gentamicin sulfate). For each replicate, 180 COCs were selected according to their morphology (Grade I and II) [19] and incubated for 22 h at 38.5 °C, 5 % CO_2_, and controlled humidity, in 100 μL drops of IVM medium (TCM 199 - GIBCO, buffered with 25 mM sodium bicarbonate, supplemented with 10 % fetal bovine serum - FBS, 0.2 mM sodium pyruvate, 50 μg/mL gentamicin sulfate, 0.5 μg/mL FSH – Folltropin, and 5 U/mL hCG), containing 20 COCs per drop, submerged in mineral oil.

### 2.2 *In vitro* fertilization

After IVM, the COCs were washed with wash media and transferred to 100 μL drops of IVF-drops medium, supplemented with 6 mg/ml BSA, 5.5 IU/ml heparin, 40 μL/ml PHE (2 mM D-penicillamine, 1 mM hypotaurine, and 245 μM epinephrine), 22 μg/ml pyruvate and 50 μg/ml gentamicin, submerged in 3 mL of mineral oil. A semen straw was previously processed in a Percoll gradient (45 % and 90 % concentration) to obtain a final concentration of 1 × 10^6^, to be added to each drop, where the oocytes were located, and incubated at 38.5 °C, 5 % of CO_2_ in controlled humidity. We carried out *in vitro* fertilization (it takes around 18 h), then the zygotes were produced. On this basis, they have ∼18 h.

### 2.3 *In vitro* culture and experimental groups

**Fig. 1:**
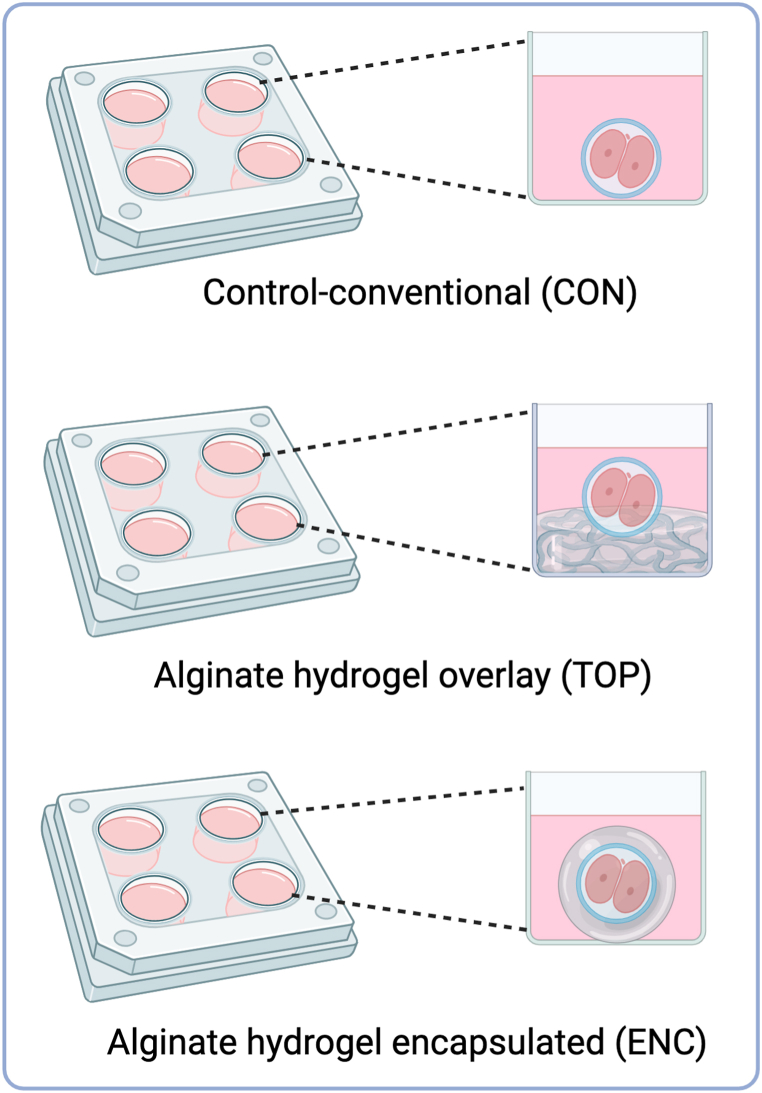
Schematic image describing the different culture systems. Experimental design demonstrating the three study groups: CON (Control-conventional); TOP (embryos cultivated on top of an 1.5 % alginate hydrogel overlay); and ENC (embryos cultivated encapsulated inside an 1.5 % alginate hydrogel sphere). Ovaries from local slaughterhouse were aspirated for COCs *in vitro* maturation (IVM), after 22 h, these COCs were *in vitro* fertilized for 18 h. Then, the presumptive zygotes were divided in the three experimental groups. They were cultivated until day 7 (D7) and finally collected for molecular or immunofluorescence analysis. Created with BioRender.com.

The presumptive zygotes were stripped all together into the IVF drops by multiple pipetting, washed using wash media and placed in their corresponding experimental groups. The three experimental groups were: 1- Control (CON); 2 - on top of an alginate surface (TOP); and 3- encapsulated inside an alginate sphere (ENC). For each replicate, 60 presumptive zygotes were divided into three groups (Fig. 1), and each group was divided into 2 wells on a 4-well plate, containing 30 presumptive zygotes per well. Each well contained 500 μL SOFaa [20] culture medium (containing 2.5 % FBS, 8 mg/ml BSA, 22 μg/ml sodium pyruvate and 50 μg/ml gentamicin) and 150 μL mineral oil. After that, the presumptive zygotes were cultured for 7 days at 38.5 °C, 5 % CO_2_, 5 % O_2_ (regulated by 90 % N_2_), and controlled humidity. On day 7 (D7), the blastocysts were classified morphologically according to Ref. [21], snap frozen in liquid nitrogen, and stored at −80 °C for further analysis. The CON group was performed conventionally, as described above. The TOP group was performed using a thin layer of 1.5 % alginate on the surface of the 4-well plate, this layer was adhered to the bottom and the embryos were cultured in conventional media on top (Supplemental file 1, 2 and 4 - Video 1 and Video 2). In the ENC group, the embryos were encapsulated inside the hydrogel formed by the 1.5 % alginate [22] and cultured in 4-well plates covered with conventional media (Supplemental file 3 and 4 - Video 3).

The following are the Supplementary data related to this article:

Video 6 Video

Video 7 Video

Video 8 Video

### 2.4 Preparation of alginate hydrogel and crosslinking solution

This experiment was carried out according to a protocol previously published by Zhao et al. (2015) for both experimental groups. For that, the alginate solution was prepared the day before the IVC. The Sodium Alginate from Sigma Aldrich-Merck (W201502) was weighed and dissolved in the SOFaaci culture medium, in order to obtain a 1.5 % solution similarly to Ref. [22]. This solution was stabilized in the culture incubator. The crosslinking solution was prepared using Mili-Q water with 50 mM CaCl_2_ and 140 mM NaCl and preheated in the incubator before cultivation. For the TOP group, 150 μL of 1.5 % alginate solution was added to the bottom of the 4-well plate and 125 μL of the crosslinking solution was slowly added to the top, after it was placed in the incubator for 6 min. Then, the 125 μL of the crosslinking solution was removed with the pipette, since a firm layer of hydrogel had already formed at the bottom of the well, and it was washed twice with the SOFaaci medium. Then, 500 μL of culture medium and 150 μL of mineral oil were added and the plate was incubated to stabilize. For the ENC group, first a 4-well plate was already stabilized with the SOFaaci medium. Then, the presumptive zygotes were placed in a drop of 1.5 % alginate and with a volume of 7 μL, 5 denuded presumptive zygotes within the 1.5 % alginate media were transferred to the crosslinking solution. In a few minutes, spheres of alginate containing embryos were solid enough for them not to fall apart. These spheres were washed in SOFaaci and placed for culture in the previously stabilized 4-well plate, with 150 μL of mineral oil on top. On D7 the spheres were broken by gently pipetting for blastocyst collections.

### 2.5 RNA isolation

From the 8 experimental routines performed, 3 pools of 10 blastocysts were obtained. Total RNA from all samples was extracted with QIAzol Lysis Reagent (Qiagen), following the manufacturer's protocol, in combination with 1.33 μL GlycoBlue co-precipitant (Thermo Fisher Scientific). The purity and concentration of the RNA was evaluated in NanoDrop One (Thermo Fisher Scientific) and the total RNA was treated with DNAse I (Invitrogen).

### 2.6 miRNA expression

In 3 pools of blastocysts per group, a profile of 383 miRNAs was analyzed. For this, cDNA was synthesized from 100 ng of total RNA with the miScript II RT kit (Qiagen), following the manufacturer's instructions, using the miScript HiSpec Buffer, selecting only for the mature miRNAs. For RT-qPCR, Power SYBR Green PCR Master Mix (Applied Biosystems), miScript Universal Primer (Qiagen), 0.05 pg cDNA per miRNA evaluated, and specific forward primers for each miRNA were used (10 μL) (Supplemental File 5 - Supplementary table 1) as described in Ref. [23]. The analysis was conducted in the QuantStudio 6 Flex (Applied Biosystems), under the following conditions: 95 °C for 15 min, followed by 45 cycles of 15 s at 94 °C, 30 s at 55 °C, and 30 s at 70 °C, and after the cycles, it was performed the melting curve analysis. Ct values greater than 37 or that had more than one peak in the melting curve were excluded. Ct values were normalized with the bta-miR-99b expression to calculate the relative expression as previously used in other manuscripts [[24], [25], [26]]. To obtain the graphs, data were transformed using 2^−ΔCt^ [27].

### 2.7 Global DNA methylation and hydroxymethylation

The blastocysts were first fixed in 4 % PFA and stored at 4 °C for further immunofluorescence analysis. Changes in global DNA methylation and hydroxymethylation were evaluated by the detection of 5-methylcytosine (5-mC) and 5-hydroxymethylcytosine (5-hmC). The embryos were incubated with PBS containing 1 % Triton X-100 for 30 min, incubated in 4N HCl for 10 min, neutralized in 100 mM Tris-HCl (pH 8.5) for 20 min. Next, the embryos were blocked in PBS with 3 % BSA and 0.3 M Glycine for 1 h. Finally, the embryos were incubated with mouse-specific anti-5-mC (Abcam; ab10805) and rabbit-anti-5-hmC (Abcam; ab214728) antibodies, diluted at a concentration of 1:1000 in PBS overnight at 4 °C. After 6 washes, the embryos were incubated with goat/anti-Mouse IgG-AlexaFluor 488 (Life Tech; A-11029), and goat/anti-rabbit IgG-AlexaFluor 594 (Life Tech; A-11012) secondary antibody for 1 h. In total, 7 blastocysts per group were analyzed by Leica SP5 confocal microscopy. For visualization of methylation excitation and emission, it was set to 488 nm and 516 nm, respectively. For hydroxymethylation excitation and emission, it was set at 543 nm and 574 nm, respectively. Confocal images of the blastocysts were captured under a 40× objective, with 3 slices at different points for each blastocyst. All images were captured under the same parameters, performing sequential acquisitions. The analyses were performed using ImageJ software, measuring the fluorescence of all blastomeres present in each image, discounting the background fluorescence.

### 2.8 RNA library preparation and sequencing

Three blastocyst pools per group (each pool containing ten blastocysts) were sequenced. RNA integrity and quantity were assessed using the RNA 6000 Pico Kit and the 2100 Bioanalyzer (Agilent Technologies). RNA sequencing (RNAseq) was performed by cDNA synthesis and amplification based on the use of the SMART-Seq HT Kit RNAseq library amplification (Takaha Bio). As per manufacturer's recommendations and depending on input RNA amount, cDNA was subjected to 19 cycles of amplification. Libraries were then prepared using the Nextera XT DNA Library Prep (Illumina) and sequenced on a NextSeq 550 (Illumina) with 75-bp single-end reads. A minimum of 10 million reads was considered per sample.

### 2.9 Global transcripts quantification and comparison among experimental groups samples

Data were visualized using R software, in which we primarily observed the classification, intensity, and difference in expression between groups. The quality of the reads was assessed using FastQC (http://www.bioinformatics.babraham.ac.uk/projects/fastqc/). The 75 bp reads were trimmed with Trimgalore and then mapped using Star [28], and identification and quantification were performed using ARS-UCD1.2 (Ensembl and NCBI) as a reference genome using the featureCounts implemented in Rsubread package [29,30] for gene count. Once the genes were identified, differential expression analysis was performed between groups using DESEq2 [31] considering a padj<0.05 and an absolute log2Folchange >1.

### 2.10 Statistical analysis

Data are presented as the mean ± standard error of the mean. The equality of the variances was tested using the Levene's test and normality was assessed with the Shapiro-Wilk test. Twelve routines were performed in total, all of them used for blastocyst rate analysis, from 8 experimental routines performed, 3 pools of 10 blastocysts were obtained (referring to different routines) for the miRNA and RNAseq analysis, each pool it is one replicate. From the other 4 routines the blastocysts obtained were fixed and 3 blastocysts were used per group from different routines for DNA methylation and hydroxymethylation analysis, and each cell from those blastocysts were analyzed using the ImageJ software. For statistical analysis of blastocyst rate, chi-square was used. To evaluate the expression of miRNAs, ANOVA was performed using the JMP7 software, followed by Tukey's test. For the analysis of global DNA methylation and hydroxymethylation it was used Kruskal-Wallis test followed by Dunn's multiple comparisons test in the GraphPad Prism 7 software. A statistical difference was considered when P < 0.05.

## 3 Results

### 3.1 Bovine embryo culture in alginate decreased blastocyst rates

**Fig. 2:**
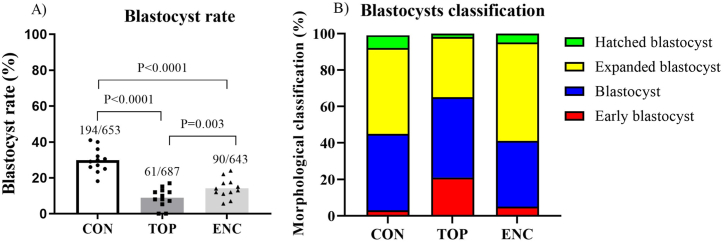
*In vitro* production rates and blastocyst developmental stages. A) Blastocyst rates in percentage after culture in CON (Control-conventional); TOP (embryos cultivated on top of an 1.5 % alginate hydrogel overlay); and ENC (embryos cultivated encapsulated inside an 1.5 % alginate hydrogel sphere). P-value was calculated using chi square test between control vs each group. B) Proportions of blastocysts in each stage.

To observe the bovine blastocyst development in different culture systems using alginate, on day 1 (D1) after *in vitro* fertilization (IVF), we divided the presumptive zygotes in three groups to be cultured until D7. Embryo culture was performed in conventional culture system (CON), on top of an alginate surface (TOP) or encapsulated inside an alginate sphere (ENC). We carried out 12 biological replicates and the blastocyst rate (Fig. 2A) was decreased (P < 0.01) in the TOP and ENC groups (8.8 % and 14 %, respectively) compared with the CON group (29.71 %). The blastocyst rate in the TOP group was also decreased compared with the ENC group (P < 0.01). Furthermore, blastocysts on day 7 were classified based on their morphology, as described by Ref. [21], so that we can observe the proportion of blastocyst development stages in the different groups (Fig. 2B).

### 3.2 Alginate culture systems altered the expression of miRNAs Bta-miR-1246, miR-1260b, and miR-541 in blastocysts

**Fig. 3:**
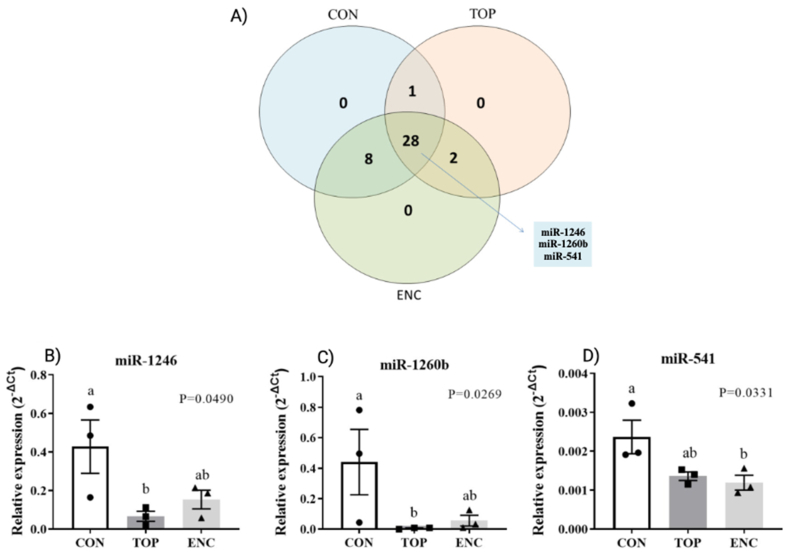
MiRNA analysis of D7 blastocysts. A) Venn diagram demonstrating the distribution of miRNAs found in blastocysts (D7) in CON (Control-conventional); TOP (embryos cultivated on top of an 1.5 % alginate hydrogel overlay); and ENC (embryos cultivated encapsulated inside an 1.5 % alginate hydrogel sphere) groups. B) miR-1246; C) miR-1260b; and D) miR-541 relative miRNAs expression in blastocysts (D7) from each experimental group (CON, TOP, and ENC). P-value was calculated using one-way ANOVA followed by Tukey. Different letters mean statistical difference (P < 0.05). Bars demonstrated the SEM.

To evaluate the effects of CON, TOP, and ENC systems in post-trancriptional regulators of gene expression such as miRNAs, we assess miRNA profile in D7 blastocysts using a custom assay. As a result, we observed three miRNAs that were differentially expressed among groups (Fig. 3A): miR-1246 and miR-1260b were downregulated in the TOP compared to the CON group (Fig. 3B–C; P < 0.05; and P < 0.05, respectively), whereas miR-541 expression was downregulated in the ENC compared to the CON group (Fig. 3D; P < 0.05).

### 3.3 Global DNA methylation and hydroxymethylation were increased in embryos cultured on top of alginate

**Fig. 4:**
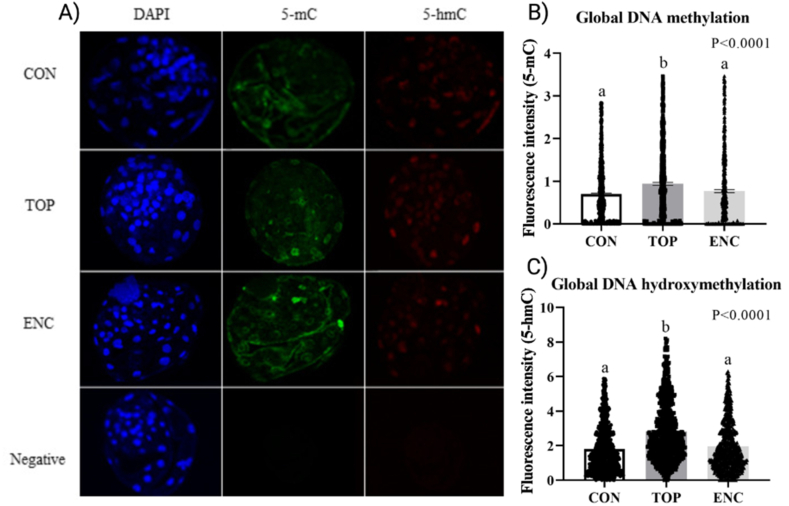
Global DNA methylation and hydroxymethylation levels in blastocysts cultured in different culture systems. A) Confocal images of blastocysts from different culture systems: CON (Control-conventional); TOP (embryos cultivated on top of an 1.5 % alginate hydrogel overlay); and ENC (embryos cultivated encapsulated inside an 1.5 % alginate hydrogel sphere). A negative control was also added to the experiment, without primary antibody. Images were acquired with 40× objectives. B) Global DNA methylation in D7 blastocysts. C) Global DNA hydroxymethylation in D7 blastocysts. Different letters represent statistical differences between groups (P < 0.05), tested by Kruskal-Wallis test followed by Dunn's multiple comparisons test. Bars indicate SEM.

To determine whether the culture environment had an effect on embryo epigenetic marks, we investigated the global levels of DNA methylation (5-methylcytosine - 5 mC) and DNA hydroxymethylation (5-hydroxymethylcytosine - 5-hmC). Our results demonstrated that both marks (Fig. 4A, B, and C) were increased in the TOP group compared with CON and ENC groups (P < 0.0001). The CON and ENC groups did not differ between them.

### 3.4 Blastocyst transcriptomes were differently modulated depending on the culture system

**Fig. 5:**
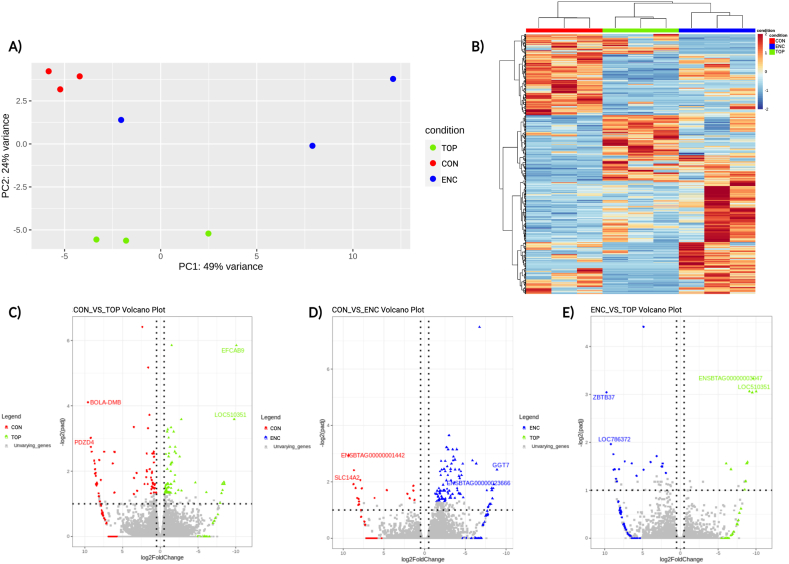
RNAseq analysis demonstrated differentially expressed genes. A) Principal component analysis (PCAs) among the groups. CON (Control-conventional); TOP (embryos cultivated on top of an 1.5 % alginate hydrogel overlay); and ENC (embryos cultivated encapsulated inside an 1.5 % alginate hydrogel sphere). B) Heatmap showing all DEGs among the groups. C) Volcano plot between the groups CON versus TOP. D) Volcano plot between the groups CON versus ENC. E) Volcano plot between the groups TOP versus ENC.

To investigate the transcriptional differences in blastocyst after their development in each culture system (CON, TOP, and ENC), we carried out RNA-seq analysis. An average of 22.397 genes out of 27.607 genes reported for the bovine genome (ARS-UCD 1.2), were identified in samples of the three groups. Analysis of the differentially expressed genes (DEGs) by principal component analysis (PCA) (Fig. 5A) and unsupervised hierarchical clustering indicated that samples had a distinct profile, clustering with each other depending on the culture system. The RNA-seq analysis revealed a total of 241 DEGs, showing a distinct expression profile between groups (Fig. 5B).

When taking the comparison between TOP versus CON, 129 genes were classified as DEGs, being 67 downregulated and 62 upregulated in the TOP group (Fig. 5C). *BOLA, DMB* and *PDZD4* had the highest fold change among downregulated ones, while *EFCAB9* and *LOC510351* were the most upregulated ones. In comparison, ENC blastocysts had 103 DEGs in relation to the CON group, with 17 genes being downregulated and 86 genes being upregulated in ENC embryos (Fig. 5D). *ENSBTAG00000001442* and *SLC14A2* had the highest fold change among downregulated ones, while *GGT7* and *ENSBTAG00000023666* were the most upregulated ones. Finally, comparison of between the two groups cultured in alginate hydrogels (TOP versus ENC) revealed 28 DEGs (Fig. 5E), where 9 genes were upregulated in the TOP group (*ZBTB37* and *LOC786372* had the highest fold change) and 19 genes were upregulated in the ENC group (*ENSBTAG00000003047* and *LOC510351* had the highest fold change). All DEGs can be found in the supplemental file 5 (Tables 2–8).

**Fig. 6:**
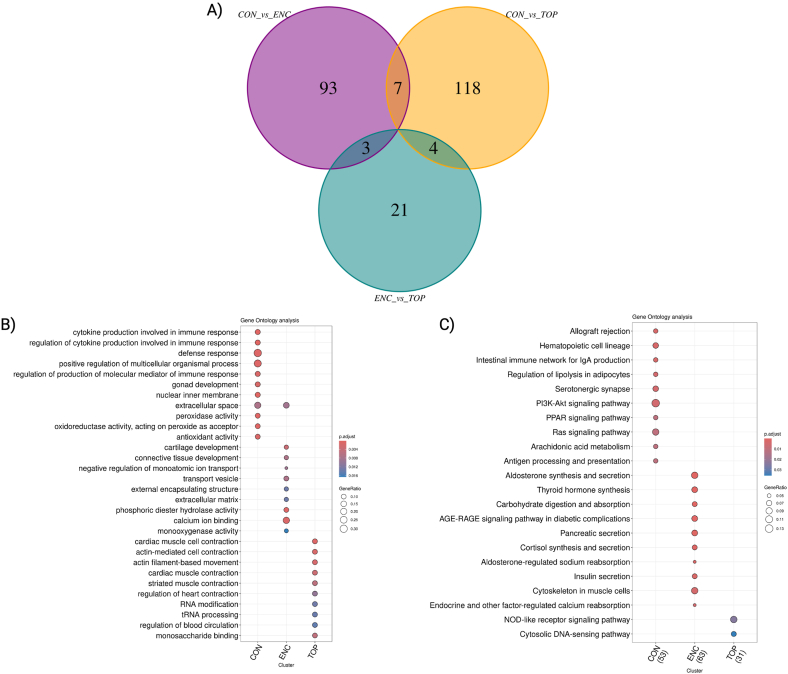
Venn diagram of DEGs and functional enrichment analysis for Gene Ontology and KEGG pathways. A) Venn diagram showing the distribution of differentially expressed genes (DEGs) across the three comparisons: CON vs ENC, CON vs TOP, and ENC vs TOP. B) Gene Ontology (GO) analysis for the DEGs from the three groups. The GO terms associated with biological processes are presented, with the size of the dots representing the gene ratio and the color indicating the adjusted p-value. C) Pathway enrichment analysis showing the most important KEGG pathways associated with DEGs across the same three conditions. The dot size reflects the gene ratio, and the color gradient represents the p-value adjustment. CON = Control-conventional group; TOP = embryos cultivated on top of an 1.5 % alginate hydrogel overlay; and ENC = embryos cultivated encapsulated inside an 1.5 % alginate hydrogel sphere.

To gain insights about the pathways altered by the culture of embryos in alginate hydrogels, we submitted the list of DEGs to the comparative cluster analysis (Fig. 6A) (supplemental file 7). As a result, we obtained 10 KEGG pathways and 11 biological process Gene Ontology (GO) for the genes differentially expressed in the CON system (Fig. 6B and C). Similarly, we identified 10 KEGG pathway and 10 biological process Gene Ontology (GO) for the genes differentially expressed in the ENC system (Fig. 6B and C). Finally, we identified 2 KEGG pathway and 10 biological process Gene Ontology (GO) for the genes differentially expressed in the TOP system (Fig. 6B and C). All KEGG pathways, biological processes, and its respective genes can be found in the supplemental file 8.

We also did a gene-by-gene search in the literature with all DEGs, seeking for critical genes involved in pluripotency, embryo development, pregnancy establishment and cell allocation. Analyzing the TOP vs CON contrast, we found genes as *POU5F1, STC1, TXNIP*, and *MEG3* upregulated in TOP, while NANOG was downregulated in the same group, showing opposite effects on crucial genes involved in pluripotency. Additionally, *ISG20* was upregulated in ENC group compared with both CON and TOP systems, *IFI44, RSAD2* and *PARM1* were upregulated in ENC compared with CON, and *IL10, PTGS1* and *ELF5* were downregulated in ENC compared with CON*.* All together, these results argue that the type of culture system can impact the RNA profile of D7 blastocysts, agreeing with other data presented in this work that TOP and ENC are similar between them compared to embryos cultured under a regular IVP condition.

Furthermore, using the TargetScan web server for predicting biological targets of miRNAs from cow genome, we investigated which genes are regulated by the 3 differentially expressed miRNAs we identified, and which of those genes were obtained as DEGs, in order to correlate both of our results [32]. The bta-miR-1246 was downregulated in the TOP group compared with the CON and it can regulate the following DEGs found to be upregulated in the TOP group compared with CON: CYP3A5, GPC6, and PRR5. The bta-miR-1260b was also downregulated in the TOP group compared with the CON and it can regulate the following DEGs found to be upregulated in the TOP group compared with CON: ASS1, CD247, CYP3A5, STC1, ARFGAP2, CNPPD1, VAMP1, RAB11FIP5 and GPC6. Additionally, the bta-miRNA-541 was found to be downregulated in the ENC group compared with the control, and this miRNA can regulate the following DEGs found to be upregulated in the ENC group compared with CON: CNP, SYT1, STC1, CTSZ, COL4A4, GYPC, SLC2A6, GPX8, TP53BP2, PARM1, CDC20B, RAB32, CREB5, ZNF664, PDGFC, RGCC, ITPKA, GLDN, ROR2, TSC22D3, and HS3ST4. These findings suggest that the downregulation of specific miRNAs, such as bta-miR-1246, bta-miR-1260b, and bta-miR-541, may contribute to the upregulation of multiple DEGs, revealing potential regulatory interactions between miRNAs and target genes in the studied conditions.

## 4 Discussion

Cell's ability to interact with its surroundings can generate physical signals as a biological response through a cascade of molecules, a phenomenon known as mechanotransduction [9,33]. Conventional *in vitro* culture (CON) is usually carried out in plastic dishes that have a greater rigidity than that found in *in vivo* tissues, such as in the reproductive tract [11]. Therefore, we hypothesized that using a softer environment during the embryo culture could improve the blastocysts development due to the physical similarity to the reproductive tract. To test this hypothesis, we cultured bovine embryos in three different systems: 1- conventional embryo culture (CON) in petri dishes, 2- embryo culture on the top of an alginate layer (TOP), and 3- embryo encapsulation in alginate spheres (ENC). Through these cultures, we evaluated the blastocyst rate, expression of a miRNA profile, DNA methylation and hydroxymethylation patterns, and the transcriptome of blastocysts produced by these three different systems. However, we observed that the blastocyst production rate dropped in cultures with alginate, being the lowest rates in the alginate TOP, thus refuting our initial hypothesis due to negative results. In addition, alginate overlay (TOP) increased the global levels of DNA methylation and hydroxymethylation. Also, TOP and ENC led to altered levels of three miRNAs and several mRNAs transcripts.

Alginate is largely used in several types of cell culture [15,16,34,35]. Alginate hydrogels, with the same alginate concentration used in our study (1.5 %), were applied in bovine blastocysts culture to study the elongation process and did not show signals of toxicity [18]. Similarly, groups of 5 embryos were encapsulated in 1.5 % sodium alginate starting from day 4 of culture and no signs of toxicity were observed, as their development rates were similar to the control group. This suggests that encapsulation on day 1 of IVF may have been a critical factor influencing the results in our study [36]. However, other research showed that encapsulating presumptive zygotes in 1 % calcium alginate also had similar blastocyst rates compared with control [35]. This suggests that the composition and concentration of alginate may have also influenced our experiments.

To investigate the molecular and epigenetic alterations caused by the *in vitro* culture in alginate hydrogels, we carried out a series of experiments. First, analysis of miRNA profile in D7 blastocysts indicated three miRNAs that were differentially expressed among the three groups: bta-miR-1246 and bta-miR-1260b had their expression reduced in blastocysts cultured in the TOP group compared to the CON and ENC culture, while miR-541 had its expression decreased in ENC blastocysts compared to the CON group. Regarding the miR-1246 function, it was found upregulated in heat-stressed sperm [37], but downregulated in extracellular vesicles secreted by blastocysts produced *in vivo* on D9 compared to *in vitro* [4], suggesting that the low levels identified in our experiments could indicate a positive effect of the culture system. Regarding the miR-1260b, it was found upregulated under hypoxia and its overexpression promoted cell proliferation in human primary pulmonary artery smooth muscle cell [38]. A similar role has been reported for miR-541, with its increased expression associating with suppression of cell proliferation and invasion in cancer cells [39]. The changes in miR-1260b and miR-541 expression, therefore, indicate that the culture system can modulate genes involved in cell proliferation through a post-transcriptional mechanism. In addition, in plasma from woman in a peri-implantation period it was showed that the miR-1260b has a negative association with basal LH level and it has also been associated with signaling pathways important for embryo development and implantation [40], indicating that this miRNA, during the embryo implantation process, may induce the activation of certain crucial genes associated with decidualization and implantation. Similarly, it was shown that the miR-1246 increases its expression by trophoblast cells after 21 days of culture, being associated with a potential role in the conceptus-endometrial cross-talk during implantation and placentation [41]. Additionally, all these altered miRNAs are predicted to regulate important pathways that are crucial for embryo development, demonstrating that alginate hydrogels can modulate the expression of these molecules. Finally, the transcriptomic changes suggest that embryos are able to respond differently to the TOP or ENC environment; although our data demonstrated a decrease in blastocyst rates, we did not test if there is an association with embryo quality.

As for the epigenetic marks, the global levels of DNA methylation and hydroxymethylation were increased in the TOP culture system. In the literature, hypermethylation is always associated with poorer developmental potential, since blastocysts produced at high oxygen tension have higher global DNA methylation than blastocysts produced in low oxygen tension [42,43], and *in vivo-*produced blastocysts have lower global DNA methylation than *in vitro-*produced ones [44]. Additionally, recent experiments involving another type of 3D culture in bovine, demonstrated an increased methylation and hydroxymethylation in liquid marbled encapsulated system compared to control group, suggesting a negative correlation between this pattern and embryo quality since this system presented lower embryo production rates [26]. Therefore, these results suggest that the TOP culture system can be associated with a lower developmental competence.

Next, we performed the transcriptomic analysis using RNAseq to determine the effects of the culture systems on gene expression. This analysis demonstrated distinct RNA profiles for blastocysts produced in the three culture systems. Analysis of DEGs demonstrated numerous genes being differently regulated in TOP compared with CON, and a large number of DEGs upregulated in ENC group in comparison with CON, meaning that alginate layers or capsule somehow induced alterations in the transcriptome of the resulting blastocysts differently. We searched the literature, gene-by-gene, looking for genes that had already been associated with important functions in D7 blastocyst or genes well-known to be regulated in the reproductive tract. As a result, we found 62 upregulated DEGs in the TOP group compared with the CON. Among them, we observed that the *POU5F1* gene (also known as *OCT4)* is strongly associated with early embryo development, being essential for blastocyst formation, pluripotency, and cell differentiation in bovine [45,46]. We also found that the *STC1* gene was upregulated in the TOP group, which has already been reported as a biomarker gene for blastocysts rate and blastocysts with better developmental kinetics [47]. Since its function is to regulate Ca^2+^ in tissues and cells [48], the observed upregulation could be a response of the embryo to the culture system and not associated with embryo quality. Similarly, *TXNIP* has an important role in apoptosis and oxidative stress. Because it was upregulated in TOP group compared with the CON, it is possibly protecting embryos from the stress caused by the hydrogels. Another developmentally important gene increased in TOP compared to CON was *MEG3,* which is highly associated with trophoblast migration, invasion, and its inhibition may be the cause of unexplained abortions [49,50]. Since it was upregulated, we can suggest that culture in hydrogels can induce changes within the trophoblast layer, which could possibly affect placentation and developmental competence.

We also found 67 genes being downregulated in blastocysts from the TOP group in contrast with CON. The most interesting gene was the *NANOG* because of its well-known essential role in forming the epiblast that will later originate the embryo itself [[51], [52], [53]], meaning that the TOP group could possibly affect the embryo growth beyond the blastocysts stage. Interestingly, the *ISG20*, which is regulated by IFNT [54,55], was increased in the ENC group compared to the CON and TOP groups. In mice, the expression of this gene in uterine epithelium can be restricted to the time of implantation [56], and its expression in the endometrium might indicate the conceptus presence in cows [54]. This implies that this system might influence the diffusion of molecules, potentially prompting the activation of genes crucial for receptivity in cows during embryonic development.

In relation to the 86 genes which were upregulated in blastocysts from ENC system compared with blastocysts from CON, we found some developmentally important genes. Among them, we found the genes: 1- *IFI44,* that is involved in blastocyst implantation [57]; 2- *RSAD2*, that had its expression increased in D8 *in vitro* blastocysts exposed to endometrial explants, indicating that it probably also has a role in embryo-maternal interaction [58]; 3- *PARM1*, that was strongly associated with blastocyst development, number of trophectoderm and inner cell mass in bovine [59]. The alterations in all these genes suggest a positive embryo response to the alginate ENC culture system. Regarding the downregulated genes, we found 17 genes decreased in blastocysts from ENC compared with CON group. One of them was the *IL10*, that may also be associated with embryo implantation, since its increase has already been observed in the blood of women 6 days after embryo transfer [60]. We also found *PTGS1* to be downregulated*.* Its expression is induced by estrogen and P4, and it may have a role in implantation in mice [61]. Finally, *ELF5* was also decreased in ENC group, and it is an important gene in trophectoderm lineage specification in mice but not in ungulates, being essential for implantation and survival of the embryo [62,63]. Thus, these results together argue that the ENC system can also induce responses in the bovine blastocyst, potentially impacting embryo quality.

According to the literature, differences in rigidity can generate mechanical stimuli that trigger biological signals, such as differences in the expression of certain genes and consequently in their protein expression [9]. Among the most studied genes are YAP and TAZ, two effectors of the Hippo signaling pathway, known to respond to various stimuli from the cellular microenvironment [64], and capable of modulate the expression of different target genes involved in cell growth and proliferation [65]. Thus, we hypothesized that we would observe differences in these mechanisms in the embryos, especially because the expression of these genes are well-established in the early embryonic phase [66,67]. However, in our RNAseq analysis, we did not observe differences in genes related to mechanotransduction pathways. Instead, we observed changes in proliferation and metabolism pathways, indicating that the alginate hydrogel can compromise the embryo development. In an attempt to investigate those negative results, osmolarity analyses were conducted. The results demonstrated that the TOP system significantly reduced IVM and IVC media osmolarity (Supplemental material file 6), which could explain the decreased blastocyst rates. Interestingly, the ENC system does not significantly alter the osmolarity of IVM or IVC media. However, although the difference was not statistically significant, the average osmolarity was higher in the ENC group, which could potentially create suboptimal conditions for the embryos, given their high sensitivity to environmental changes. Therefore, while our findings have pushed further our understanding of the effects of this type of cell culture system on *in vitro* embryo production, further experiments with different experimental designers would be interesting to fully address the remaining questions.

## 5 Conclusion

Our outcomes show that TOP and ENC treatments had different effects on miRNAs associated with cell proliferation. We also observed higher levels of global DNA methylation and hydroxymethylation in the TOP group compared to the CON and ENC groups. Additionally, RNA sequencing (RNA-seq) analysis showed differences in several developmentally important genes between the groups. Overall, these findings show that, while blastocysts could be produced (although in smaller percentages than in control conditions), the alginate hydrogel applied in the experiment had a negative effect on bovine embryo development. This observation is supported by changes in global epigenetic marks in embryos cultured on the alginate layer, as well as changes in the transcriptome of blastocysts produced on or encapsulated in the alginate hydrogel. Our data interpretation suggests caution in the use of alginate hydrogels before we fully understand their effects on the *in vitro* produced embryos. Moreover, our osmolarity analyses showed that the hydrogels may have created a suboptimal environment for *in vitro* production of bovine embryos. Furthermore, new experimental conditions can be investigated, such as different concentrations of alginate in hydrogels, changes in incubation times, alterations in the crosslinking solution, among others.

## CRediT authorship contribution statement

**Giuliana de A. Ferronato:** Writing – review & editing, Writing – original draft, Methodology, Investigation, Formal analysis, Data curation, Conceptualization. **Paola M. da S. Rosa:** Methodology, Investigation. **Alessandra Bridi:** Methodology, Investigation. **Angélica Camargo dos Santos:** Formal analysis, Data curation. **Ricardo P. Nociti:** Formal analysis, Data curation. **Marcos Roberto Chiaratti:** Formal analysis, Data curation. **Felipe Perecin:** Methodology, Investigation. **Flávio V. Meirelles:** Methodology, Investigation. **Juliano R. Sangalli:** Writing – review & editing, Methodology, Investigation, Formal analysis, Data curation, Conceptualization. **Juliano C. da Silveira:** Writing – review & editing, Writing – original draft, Methodology, Investigation, Funding acquisition, Formal analysis, Data curation, Conceptualization.

## Ethics statement

All experiments conducted in this study were approved by the University of São Paulo Research Ethics Committee (protocol number: 5343150721).

## Data availability statement

Original data are available from the corresponding author upon reasonable request. RNAseq data is available at NCBI accession number: GSE249413.

## Funding

The authors would like to thank the 10.13039/501100003593National Council for Scientific and Technological Development – CNPq (grant number #420152/2018-0) and research fellowship of J.C.S (308101/2021-9); the São Paulo Research Foundation - 10.13039/501100001807FAPESP (grant number #2014/22887-0; #2019/25675-7; #2021/06645-0); this study was financed in part by the Coordenação de Aperfeiçoamento de Pessoal de Nível Superior - Brasil (10.13039/501100002322CAPES) - Finance Code 001.

## Declaration of competing interest

The authors declare that they have no known competing financial interests or personal relationships that could have appeared to influence the work reported in this paper.
