# Supplementary material for: Transcriptomic effects of alginate hydrogel applied to the production of bovine embryos
Source: Heliyon. 2024 Dec 6;10(24):e40957. doi: 10.1016/j.heliyon.2024.e40957 (PMC11700250; doi:10.1016/j.heliyon.2024.e40957)
Supplement: Multimedia component 1 [file mmc1.docx]

SM Video 1 - Preparation of the TOP Group: Addition of 150 µl of 1.5% alginate in SOF medium on the surface of the plate, followed by 125 µl of crosslinking solution, and left for 6 minutes in the incubator.

SM Video 2 - 125 µl of the crosslinking solution was removed, and embryo culture medium was added.

SM Video 3 - Preparation of the Encapsulated Group: Addition of 1.5% alginate containing the embryos to the crosslinking solution, followed by transfer to the medium in the culture plate.
