# Supplementary material for: Transcriptomic effects of alginate hydrogel applied to the production of bovine embryos
Source: Heliyon. 2024 Dec 6;10(24):e40957. doi: 10.1016/j.heliyon.2024.e40957 (PMC11700250; doi:10.1016/j.heliyon.2024.e40957)
Supplement: Multimedia component 2 [file mmc2.docx]

**Supplementary Table 1**. MicroRNAs primers sequences used in quantitative RT-PCR analysis.

| **miRNA** | **Sequence** | **MiRNA** | **Sequence** |
| --- | --- | --- | --- |
| bta-let-7a-3p | CTATACAATCTACTGTCTTTC | bta-miR-33b | GTGCATTGCTGTTGCATTGC |
| bta-miR-103 | AGCAGCATTGTACAGGGCTATGA | bta-miR-340 | TCCGTCTCAGTTACTTTATAGCC |
| bta-let-7a-5p | TGAGGTAGTAGGTTGTATAGTT | bta-miR-365-3p | TAATGCCCCTAAAAATCCTTAT |
| bta-miR-105a | TCAAATGCTCAGACTCCTGTGGT | bta-miR-342 | TCTCACACAGAAATCGCACCCATCT |
| bta-let-7b | TGAGGTAGTAGGTTGTGTGGTT | bta-miR-365-5p | AGGGACTTTTGGGGGCAGATGTG |
| bta-miR-105b | TCAAATGCTCAGACTCCTTGGT | bta-miR-345-3p | CCTGAACTAGGGGTCTGGAG |
| bta-let-7c | TGAGGTAGTAGGTTGTATGGTT | bta-miR-367 | GAATTGCACTTTAGCAATGGTGA |
| bta-miR-106a | AAAAGTGCTTACAGTGCAGGTA | bta-miR-345-5p | GCTGACTCCTAGTCCAGTGCT |
| bta-let-7d | AGAGGTAGTAGGTTGCATAGTT | bta-miR-369-3p | AATAATACATGGTTGATCTTT |
| bta-miR-106b | TAAAGTGCTGACAGTGCAGAT | bta-miR-346 | TGTCTGCCCGCATGCCTGCCTCT |
| bta-let-7e | TGAGGTAGGAGGTTGTATAGT | bta-miR-369-5p | ATCGACCGTGTTATATTCGC |
| bta-miR-107 | AGCAGCATTGTACAGGGCTATC | bta-miR-34a | TGGCAGTGTCTTAGCTGGTTGT |
| bta-let-7f | TGAGGTAGTAGATTGTATAGTT | bta-miR-370 | GCCTGCTGGGGTGGAACCTGGT |
| bta-miR-10a | TACCCTGTAGATCCGAATTTGTG | bta-miR-34b | AGGCAGTGTAATTAGCTGATTG |
| bta-let-7g | TGAGGTAGTAGTTTGTACAGTT | bta-miR-371 | AAGTGCCGCCATGTTTTGAGTGT |
| bta-miR-10b | TACCCTGTAGAACCGAATTTGTG | bta-miR-34c | AGGCAGTGTAGTTAGCTGATTG |
| bta-let-7i | TGAGGTAGTAGTTTGTGCTGTT | bta-miR-374a | TTATAATACAACCTGATAAGTG |
| bta-miR-122 | TGGAGTGTGACAATGGTGTTTG | bta-miR-361 | TTATCAGAATCTCCAGGGGTAC |
| bta-miR-1 | TGGAATGTAAAGAAGTATGTAT | bta-miR-374b | ATATAATACAACCTGCTAAGTG |
| bta-miR-124a | TAAGGCACGCGGTGAATGCCAAG | bta-miR-362-3p | AACACACCTATTCAAGGATTC |
| bta-miR-100 | AACCCGTAGATCCGAACTTGTG | bta-miR-375 | TTTTGTTCGTTCGGCTCGCGTGA |
| bta-miR-124b | TAAGGCACGCGGTGAATGCCAAG | bta-miR-362-5p | AATCCTTGGAACCTAGGTGTGAGT |
| bta-miR-101 | TACAGTACTGTGATAACTGAA | bta-miR-376a | ATCATAGAGGAAAATCCACGT |
| bta-miR-125a | TCCCTGAGACCCTTTAACCTGTG | bta-miR-363 | ATTGCACGGTATCCATCTGCG |
| bta-miR-125b | TCCCTGAGACCCTAACTTGTGA | bta-miR-376b | ATCATAGAGGAAAATCCATGTT |
| bta-miR-133b | TTTGGTCCCCTTCAACCAGCTA | bta-miR-376c | GTGGATATTCCTTCTATGTTTA |
| bta-miR-126-3p | CGTACCGTGAGTAATAATGCG | bta-miR-382 | GAAGTTGTTCGTGGTGGATTCG |
| bta-miR-133c | ATTTGGTTCCATTTTACCAGC | bta-miR-376d | ATCATAGAGGAAAATCCACAT |
| bta-miR-126-5p | CATTATTACTTTTGGTACGCG | bta-miR-383 | AGATCAGAAGGTGATTGTGGCT |
| bta-miR-134 | TGTGACTGGTTGACCAGAGTGG | bta-miR-376e | AACATAGAGGAAAATCCACATT |
| bta-miR-127 | TCGGATCCGTCTGAGCTTGGCT | bta-miR-409a | AGGTTACCCGAGCAACTTTGCAT |
| bta-miR-135a | TATGGCTTTTTATTCCTATGTGA | bta-miR-377 | ATCACACAAAGGCAACTTTTGT |
| bta-miR-128 | TCACAGTGAACCGGTCTCTTT | bta-miR-409b | GGGGTTCACCGAGCAACATTC |
| bta-miR-135b | TATGGCTTTTCATTCCTATGTGA | bta-miR-378 | ACTGGACTTGGAGTCAGAAGGC |
| bta-miR-129 | CTTTTTGCGGTCTGGGCTTGCT | bta-miR-410 | AATATAACACAGATGGCCTGT |
| bta-miR-136 | ACTCCATTTGTTTTGATGATGGA | bta-miR-378b | ACTTGACTTGGAGTCAGAAGGC |
| bta-miR-129-3p | AAGCCCTTACCCCAAAAAGCAT | bta-miR-411a | ATAGTAGACCGTATAGCGTACG |
| bta-miR-137 | TTATTGCTTAAGAATACGCGTAG | bta-miR-378c | ACTGGACTTGGAGTCAGAAGT |
| bta-miR-129-5p | CTTTTTGCGGTCTGGGCTTGCT | bta-miR-411b | TGGTCGACCATAAAACGTACGT |
| bta-miR-138 | AGCTGGTGTTGTGAATCAGGCCG | bta-miR-378d | CTGGACTTGGAGTCAGAAGACC |
| bta-miR-130a | CAGTGCAATGTTAAAAGGGCAT | bta-miR-411c-3p | TGTATGTCAACTGATCCACAGT |
| bta-miR-139 | TCTACAGTGCACGTGTCTCCAGT | bta-miR-379 | TGGTAGACTATGGAACGTAGG |
| bta-miR-130b | CAGTGCAATGATGAAAGGGCAT | bta-miR-411c-5p | GGTTGATCAGAGAACATACATT |
| bta-miR-140 | TACCACAGGGTAGAACCACGGA | bta-miR-380-3p | TATGTAATGTGGTCCACGTCT |
| bta-miR-132 | TAACAGTCTACAGCCATGGTCG | bta-miR-412 | ACTTCACCTGGTCCACTAGCTGT |
| bta-miR-141 | TAACACTGTCTGGTAAAGATGG | bta-miR-380-5p | TGGTTGACCATAGAACATGCGC |
| bta-miR-133a | TTTGGTCCCCTTCAACCAGCTG | bta-miR-421 | ATCAACAGACATTAATTGGGCGC |
| bta-miR-142-3p | AGTGTTTCCTACTTTATGGATG | bta-miR-381 | TATACAAGGGCAAGCTCTCTGT |
| bta-miR-142-5p | CATAAAGTAGAAAGCACTAC | bta-miR-423-3p | AAGCTCGGTCTGAGGCCCCTCAGT |
| bta-miR-151-3p | CTAGACTGAAGCTCCTTGAGG | bta-miR-423-5p | TGAGGGGCAGAGAGCGAGACTTT |
| bta-miR-143 | TGAGATGAAGCACTGTAGCTCG | bta-miR-449c | AGGCAGTGCATCTCTAGCTGG |
| bta-miR-151-5p | TCGAGGAGCTCACAGTCTAGT | bta-miR-424-3p | CAAAACGTGAGGCGCTGCTAT |
| bta-miR-144 | TACAGTATAGATGATGTACTAG | bta-miR-449d | GAAGGCTGTGTGCTGTGGAG |
| bta-miR-152 | TCAGTGCATGACAGAACTTGGG | bta-miR-424-5p | CAGCAGCAATTCATGTTTTGA |
| bta-miR-145 | GTCCAGTTTTCCCAGGAATCCCT | bta-miR-450a | TTTTGCGATGTGTTCCTAATAT |
| bta-miR-153 | TTGCATAGTCACAAAAGTGATC | bta-miR-425-3p | ATCGGGAATGTCGTGTCCGCCC |
| bta-miR-146a | TGAGAACTGAATTCCATAGGTTGT | bta-miR-450b | TTTTGCAATATGTTCCTGAATA |
| bta-miR-154a | TAGGTTATCCGTGTAGCCTTCG | bta-miR-425-5p | ATGACACGATCACTCCCGTTGA |
| bta-miR-146b | TGAGAACTGAATTCCATAGGCTGT | bta-miR-451 | AAACCGTTACCATTACTGAGTTT |
| bta-miR-154b | AGAGGTCTTCCATGGTGCATTCG | bta-miR-429 | TAATACTGTCTGGTAATGCCGT |
| bta-miR-147 | GTGTGCGGAAATGCTTCTGCTA | bta-miR-452 | TGTTTGCAGAGGAAACTGAGAC |
| bta-miR-154c | AGATATTGCACGGTTGATCTCT | bta-miR-431 | TGTCTTGCAGGCCGTCATGCAGG |
| bta-miR-148a | TCAGTGCACTACAGAACTTTGT | bta-miR-4523 | GACCGAGAGGGCCTCGGCTGT |
| bta-miR-155 | TTAATGCTAATCGTGATAGGGGT | bta-miR-432 | TCTTGGAGTAGGTCATTGGGTGG |
| bta-miR-148b | TCAGTGCATCACAGAACTTTGT | bta-miR-453 | AGGTTGTCCGTGGTGAGTTCGCA |
| bta-miR-15a | TAGCAGCACATAATGGTTTGT | bta-miR-433 | ATCATGATGGGCTCCTCGGTGT |
| bta-miR-149-3p | GAGGGAGGGACGGGGGCTGTGC | bta-miR-454 | TAGTGCAATATTGCTTATAGGGT |
| bta-miR-15b | TAGCAGCACATCATGGTTTACA | bta-miR-448 | TTGCATATGTAGGATGTCCCAT |
| bta-miR-149-5p | TCTGGCTCCGTGTCTTCACTCCC | bta-miR-455-3p | GCAGTCCATGGGCATATACACT |
| bta-miR-16a | TAGCAGCACGTAAATATTGGTG | bta-miR-449a | TGGCAGTGTATTGTTAGCTGGT |
| bta-miR-150 | TCTCCCAACCCTTGTACCAGTGT | bta-miR-455-5p | TATGTGCCTTTGGACTACATC |
| bta-miR-16b | TAGCAGCACGTAAATATTGGC | bta-miR-449b | AGGCAGTGTATTGTTAGCTGGC |
| bta-miR-17-3p | ACTGCAGTGAAGGCACTTGT | bta-miR-483 | TCACTCCTCTCCTCCCGTCTT |
| bta-miR-188 | CATCCCTTGCATGGTGGAGGGT | bta-miR-484 | TCAGGCTCAGTCCCCTCCCGAT |
| bta-miR-17-5p | CAAAGTGCTTACAGTGCAGGTAGT | bta-miR-496 | TGAGTATTACATGGCCAATCTC |
| bta-miR-18a | TAAGGTGCATCTAGTGCAGATA | bta-miR-485 | AGAGGCTGGCCGTGATGAATTCG |
| bta-miR-181a | AACATTCAACGCTGTCGGTGAGTT | bta-miR-497 | CAGCAGCACACTGTGGTTTGTA |
| bta-miR-18b | TAAGGTGCATCTAGTGCAGTTA | bta-miR-486 | TCCTGTACTGAGCTGCCCCGAG |
| bta-miR-181b | AACATTCATTGCTGTCGGTGGGTT | bta-miR-499 | TTAAGACTTGCAGTGATGTTT |
| bta-miR-190a | TGATATGTTTGATATATTAGGT | bta-miR-487a | AATCATACAGGGACATCCAGT |
| bta-miR-181c | AACATTCAACCTGTCGGTGAGTTT | bta-miR-500 | TAATCCTTGCTACCTGGGTGAGA |
| bta-miR-190b | TGATATGTTTGATATTGGGTT | bta-miR-487b | AATCGTACAGGGTCATCCACTT |
| bta-miR-181d | AACATTCATTGTTGTCGGTGGGT | bta-miR-502a | AATGCACCTGGGCAAGGATTCA |
| bta-miR-191 | CAACGGAATCCCAAAAGCAGCTG | bta-miR-488 | TTGAAAGGCTGTTTCTTGGTC |
| bta-miR-182 | TTTGGCAATGGTAGAACTCACACT | bta-miR-502b | AATCCACCTGGGCAAGGATTC |
| bta-miR-192 | CTGACCTATGAATTGACAGCCAG | bta-miR-489 | GTGACATCACATATATGGCGAC |
| bta-miR-183 | TATGGCACTGGTAGAATTCACTG | bta-miR-503-3p | GGAGTATTGTTTCTGCTGCCCGG |
| bta-miR-193a | GGGACTTTGTAGGCCAGTT | bta-miR-490 | CAACCTGGAGGACTCCATGCTG |
| bta-miR-184 | TGGACGGAGAACTGATAAGGGT | bta-miR-503-5p | TAGCAGCGGGAACAGTACTG |
| bta-miR-193a-3p | AACTGGCCTACAAAGTCCCAGT | bta-miR-491 | AGTGGGGAACCCTTCCATGAGG |
| bta-miR-185 | TGGAGAGAAAGGCAGTTCCTGA | bta-miR-504 | AGACCCTGGTCTGCACTCTGTC |
| bta-miR-193a-5p | TGGGTCTTTGCGGGCGAGATGA | bta-miR-493 | TGAAGGTCTACTGTGTGCCAGG |
| bta-miR-186 | CAAAGAATTCTCCTTTTGGGCT | bta-miR-505 | CGTCAACACTTGCTGGTTTCCT |
| bta-miR-193b | AACTGGCCCACAAAGTCCCGCTTT | bta-miR-494 | TGAAACATACACGGGAAACCTC |
| bta-miR-187 | TCGTGTCTTGTGTTGCAGCCGG | bta-miR-532 | CATGCCTTGAGTGTAGGACCGT |
| bta-miR-194 | TGTAACAGCAACTCCATGTGGA | bta-miR-495 | AAACAAACATGGTGCACTTCTT |
| bta-miR-195 | TAGCAGCACAGAAATATTGGCA | bta-miR-539 | GGAGAAATTATCCTTGGTGTGT |
| bta-miR-200c | TAATACTGCCGGGTAATGATGGA | bta-miR-541 | TGGTGGGCACAGAATCCGGCCT |
| bta-miR-196a | TAGGTAGTTTCATGTTGTTGGG | bta-miR-582 | TTACAGTTGTTCAACCAGTTACT |
| bta-miR-202 | TTCCTATGCATATACTTCTTT | bta-miR-542-5p | TCGGGGATCATCATGTCACGAG |
| bta-miR-196b | TAGGTAGTTTCCTGTTGTTGGGA | bta-miR-584 | TGGTTTGCCTGGGACTGAG |
| bta-miR-204 | TTCCCTTTGTCATCCTATGCCT | bta-miR-543 | AAACATTCGCGGTGCACTTCTT |
| bta-miR-197 | TTCACCACCTTCTCCACCCAGC | bta-miR-592 | ATTGTGTCAATATGCGATGATGT |
| bta-miR-205 | TCCTTCATTCCACCGGAGTCTG | bta-miR-544a | ATTCTGCATTTTTAGCAAGTTC |
| bta-miR-199a-3p | ACAGTAGTCTGCACATTGGTTA | bta-miR-599 | GTTGTGTCAGTTTATCAAAC |
| bta-miR-206 | TGGAATGTAAGGAAGTGTGTGG | bta-miR-544b | ATTCTGCATTTCTAACAAGTTC |
| bta-miR-199a-5p | CCCAGTGTTCAGACTACCTGTT | bta-miR-615 | GGGGGTCCCCGGTGCTCGGATC |
| bta-miR-208a | ATAAGACGAGCAAAAAGCTTGT | bta-miR-545-3p | ATCAACAAACATTTATTGTGTG |
| bta-miR-199b | CCCAGTGTTTAGACTATCTGTTC | bta-miR-628 | ATGCTGACATATTTACTAGAGG |
| bta-miR-208b | ATAAGACGAACAAAAGGTTTGT | bta-miR-545-5p | TCAGTAAATGTTTATTGGATG |
| bta-miR-199c | TACAGTAGTCTGCACATTGG | bta-miR-631 | AGACCTGGCTTAGACCTCAGC |
| bta-miR-20a | TAAAGTGCTTATAGTGCAGGTAG | bta-miR-551a | GCGACCCAATCTTGGTTTCCA |
| bta-miR-19a | TGTGCAAATCTATGCAAAACTGA | bta-miR-652 | AATGGCGCCACTAGGGTTGTG |
| bta-miR-20b | CAAAGTGCTCACAGTGCAGGTA | bta-miR-551b | GGCGACCCATACTTGGTTTCAG |
| bta-miR-19b | TGTGCAAATCCATGCAAAACTGA | bta-miR-653 | GTGTTGAAACAATCTCTGTTG |
| bta-miR-21-3p | AACAGCAGTCGATGGGCTGTCT | bta-miR-562 | AAAGCAGCTGTACCATTTAC |
| bta-miR-200a | TAACACTGTCTGGTAACGATGTT | bta-miR-654 | TATGTCTGCTGACCATCACCTT |
| bta-miR-21-5p | TAGCTTATCAGACTGATGTTGACT | bta-miR-568 | ATGTATAAATGTATACACAC |
| bta-miR-200b | TAATACTGCCTGGTAATGATG | bta-miR-655 | ATAATACATGGTTAACCTCTCT |
| bta-miR-210 | ACTGTGCGTGTGACAGCGGCTGA | bta-miR-574 | TGAGTGTGTGTGTGTGAGTGTGTG |
| bta-miR-211 | TTCCCTTTGTCATCCTTTGCC | bta-miR-656 | AATATTATACAGTCAACCTCT |
| bta-miR-22-5p | AGTTCTTCAGTGGCAAGCTTTA | bta-miR-658 | GGCGGAGGGAAGCGGGTCCGTTGGT |
| bta-miR-212 | ACCTTGGCTCTAGACTGCTTACT | bta-miR-758 | TTTGTGACCTGGTCCACTAACC |
| bta-miR-221 | AGCTACATTGTCTGCTGGGTTT | bta-miR-660 | TACCCATTGCATATCGGAGCTG |
| bta-miR-214 | ACAGCAGGCACAGACAGGCAGT | bta-miR-759 | GCAGACTGCAAACAATTTTGAC |
| bta-miR-222 | AGCTACATCTGGCTACTGGGT | bta-miR-664a | CAGGCTGGGGTGTGTGTGGATG |
| bta-miR-215 | ATGACCTATGAATTGACAGACA | bta-miR-760-3p | CGGCTCTGGGTCTGTGGGGA |
| bta-miR-223 | TGTCAGTTTGTCAAATACCCCA | bta-miR-664b | TATTCATTTATCTCCCAGCCTAC |
| bta-miR-216a | TAATCTCAGCTGGCAACTGTGA | bta-miR-760-5p | CCCCTCAGTCCACCAGAGCCCG |
| bta-miR-224 | CAAGTCACTAGTGGTTCCGTTTA | bta-miR-665 | ACCAGTAGGCCGAGGCCCCT |
| bta-miR-216b | AAATCTCTGCAGGCAAATGTGA | bta-miR-761 | GCAGCAGGGTGAAACTGACACA |
| bta-miR-23a | ATCACATTGCCAGGGATTTCCA | bta-miR-669 | TGTGGGTGTGTGCATGTGCGTG |
| bta-miR-217 | TACTGCATCAGGAACTGATTGGAT | bta-miR-763 | CCAGCTGGGAGGAACCAGTGGC |
| bta-miR-23b-3p | ATCACATTGCCAGGGATTACCAC | bta-miR-670 | TCCCTGAGTATATGTGGTGAA |
| bta-miR-218 | TTGTGCTTGATCTAACCATGTG | bta-miR-764 | GGTGCTCACTCGTCCTTCT |
| bta-miR-23b-5p | GGGTTCCTGGCATGCTGATTT | bta-miR-671 | AGGAAGCCCTGGAGGGGCTGGAG |
| bta-miR-219 | AGAGTTGAGTCTGGACGTCCCG | bta-miR-767 | TGCACCATGGTTGTCTGAGCATG |
| bta-miR-24 | GTGCCTACTGAGCTGATATCAGT | bta-miR-677 | CTCACTGATGAGCAGCTTCTGAC |
| bta-miR-219-3p | AGAATTGTGGCTGGACATCTG | bta-miR-769 | TGAGACCTCCGGGTTCTGAGCT |
| bta-miR-24-3p | TGGCTCAGTTCAGCAGGAACAG | bta-miR-7 | TGGAAGACTAGTGATTTTGTTGTT |
| bta-miR-219-5p | TGATTGTCCAAACGCAATTCTT | bta-miR-873 | GCAGGAACTTGTGAGTCTCCT |
| bta-miR-25 | CATTGCACTTGTCTCGGTCTGA | bta-miR-708 | AAGGAGCTTACAATCTAGCTGGG |
| bta-miR-22-3p | AAGCTGCCAGTTGAAGAACTG | bta-miR-874 | CTGCCCTGGCCCGAGGGACCGA |
| bta-miR-26a | TTCAAGTAATCCAGGATAGGCT | bta-miR-744 | TGCGGGGCTAGGGCTAACAGCA |
| bta-miR-26b | TTCAAGTAATTCAGGATAGGTT | bta-miR-875 | TATACCTCAGTTTTATCAGGTG |
| bta-miR-29d-3p | TAGCACCATTTGAAATCGATTA | bta-miR-876 | TGGATTTCTTTGTGAATCACCA |
| bta-miR-26c | AGCCTATCCTGGATTACTTGAA | bta-miR-98 | TGAGGTAGTAAGTTGTATTGTT |
| bta-miR-29d-5p | TGACCGATTTCTCCTGGTGTT | bta-miR-877 | GTAGAGGAGATGGCGCAGGG |
| bta-miR-27a-3p | TTCACAGTGGCTAAGTTCCG | bta-miR-99a-3p | CAAGCTCGCTTCTATGGGT |
| bta-miR-29e | TAGCATCATTTGAAATCAGTGTTT | bta-miR-885 | TCCATTACACTACCCTGCCTCT |
| bta-miR-27a-5p | AGGGCTTAGCTGCTTGTGAGCA | bta-miR-99a-5p | AACCCGTAGATCCGATCTTGT |
| bta-miR-301a | CAGTGCAATAGTATTGTCAAAGCAT | bta-miR-9-3p | ATAAAGCTAGATAACCG |
| bta-miR-27b | TTCACAGTGGCTAAGTTCTGC | bta-miR-99b | CACCCGTAGAACCGACCTTGCG |
| bta-miR-301b | CAGTGCAATGATATTGTCAAAGCAT | bta-miR-9-5p | TCTTTGGTTATCTAGCTGTATG |
| bta-miR-28 | AAGGAGCTCACAGTCTATTGAG | bta-miR-1179 | AAGCATTCTTTCATTGGTTGG |
| bta-miR-302a | AAGTGCTTCCATGTTTTAGTGA | bta-miR-92a | TATTGCACTTGTCCCGGCCTGT |
| bta-miR-296-3p | GAGGGTTGGGCGGAGGCTTTCC | bta-miR-1185 | AGAGGATACCCTTTGTATGTT |
| bta-miR-302b | TAAGTGCTTCCATGTTTTAGTAG | bta-miR-92b | TATTGCACTCGTCCCGGCCTCC |
| bta-miR-296-5p | GAGGGCCCCCCCCAATCCT | bta-miR-1193 | TAGGTCACCCGTTTGACTATC |
| bta-miR-302c | TAAGTGCTTCCATGTTTCAGTGG | bta-miR-93 | CAAAGTGCTGTTCGTGCAGGTA |
| bta-miR-299 | TGGTTTACCGTCCCACATACAT | bta-miR-1197 | TAGGACACATGGTCTACTTCT |
| bta-miR-302d | TAAGTGCTTCCATGTTTTAGT | bta-miR-935 | CCAGTTACCGCTTCCGCTACCGC |
| bta-miR-29a | CTAGCACCATCTGAAATCGGTTA | bta-miR-122 | TGGAGTGTGACAATGGTGTTTG |
| bta-miR-3064 | TTGCCACACTGCAACACCTTACA | bta-miR-940 | AAGGCTGGGCCCCCGCTCCGC |
| bta-miR-29b | TAGCACCATTTGAAATCAGTGTT | bta-miR-1224 | GTGAGGACTCGGGAGGTGGAG |
| bta-miR-30a-5p | TGTAAACATCCTCGACTGGAAGCT | bta-miR-95 | TTCAACGGGTATTTATTGAGCA |
| bta-miR-29c | TAGCACCATTTGAAATCGGTTA | bta-miR-1225-3p | CCGAGCCCCTGTGCCGCCCCCAG |
| bta-miR-30b-3p | CTGGGAGGTGGATGTTTACTT | bta-miR-96 | TTTGGCACTAGCACATTTTTGCT |
| bta-miR-30b-5p | TGTAAACATCCTACACTCAGCT | bta-miR-1246 | AATGGATTTTTGGAGCAGG |
| bta-miR-328 | CTGGCCCTCTCTGCCCTTCCGT | bta-miR-1247-3p | CGGGAACGTCGGGACTGGAGC |
| bta-miR-30c | TGTAAACATCCTACACTCTCAGC | bta-miR-1296 | TTAGGGCCCTGGCTCCATCTCC |
| bta-miR-329a | AACACACCTGGTTAACCTTTTT | bta-miR-1247-5p | ACCCGTCCCGTGCGTCCCCGGA |
| bta-miR-30d | TGTAAACATCCCCGACTGGAAGCT | bta-miR-1298 | TTCATTCGGCTGTCCAGATGTA |
| bta-miR-329b | AGAGGTTTTCTGGGTTTCTGTTT | bta-miR-1248 | ACCTTCTTGTATAAGCACTGTGCTAAA |
| bta-miR-30e-5p | TGTAAACATCCTTGACTGGAAGCT | bta-miR-1301 | TTGCAGCTGCCTAGGAGTGATTTC |
| bta-miR-330 | GCAAAGCACACGGCCTGCAGAGA | bta-miR-1249 | ACGCCCTTCCCCCCCTTCTTCA |
| bta-miR-30f | TGTAAACACCCTACACTCTCAGCT | bta-miR-1306 | CCACCTCCCCTGCAAACGTCC |
| bta-miR-331-3p | GCCCCTGGGCCTATCCTAGAA | bta-miR-1260b | ATCCCACCACTGCCACCA |
| bta-miR-31 | AGGCAAGATGCTGGCATAGCT | bta-miR-1307 | ACTCGGCGTGGCGTCGGTCGTG |
| bta-miR-331-5p | TCTAGGTATGGTCCCAGG | bta-miR-1271 | CTTGGCACCTAGTAAGTACTCA |
| bta-miR-32 | TATTGCACATGACTAAGTTGCAT | bta-miR-1343-3p | CTCCTGGGGCCCGCACTCTC |
| bta-miR-335 | TCAAGAGCAATAACGAAAAATGT | bta-miR-1277 | TACGTAGATATATATGTATTTT |
| bta-miR-320a | AAAAGCTGGGTTGAGAGGGCGA | bta-miR-1343-5p | TGGGGAGCGGCCCCCGGGCGGG |
| bta-miR-338 | TCCAGCATCAGTGATTTTGTTGA | bta-miR-1281 | TCGCCTCCTCCTCTCCC |
| bta-miR-320b | AGCTGGGTTGAGAGGGTGGT | bta-miR-1388-3p | ATCTCAGGTTTGTCAGCCCGCA |
| bta-miR-339a | TCCCTGTCCTCCAGGAGCTCAC | bta-miR-1282 | TCGTTTGCCTTTTTCTGCTT |
| bta-miR-323 | GCACATTACACGGTCGACCTCT | RNT43 snoRNA | CTTATTGACGGGCGGACAGAAAC |
| bta-miR-339b | TCCCTGTCCTCCAGGAGCTC | bta-miR-1284 | TCTGCACAGACCCTGGCTTTTC |
| bta-miR-324 | CGCATCCCCTAGGGCATTGGTGT | Hm/Ms/Rt T1 snRNA | CGACTGCATAATTTGTGGTAGTGG |
| bta-miR-33a | GTGCATTGTAGTTGCATTGCA | bta-miR-1287 | TGCTGGATCAGTGGTTTGAGTC |
| bta-miR-326 | CCTCTGGGCCCTTCCTCCAG | bta-miR-1291 | TGGCCCTGACTGAAGACCTGCAGT |

**Supplementary table 2.** DEGs upregulated in TOP group compared with CON group.

| ENTREZ_GENE_ID | Gene Symbol | Log2FoldChange | p-adjusted |
| --- | --- | --- | --- |
| 280726 | ASS1 | -1.28182123369624 | 0.00181334586052221 |
| 281056 | CD247 | -4.64984767773921 | 0.0223350608695638 |
| 281409 | PLOD1 | -2.07232651603002 | 0.00287795461712212 |
| 282015 | PSMC5 | -0.583564845835522 | 0.049868331179072 |
| 282214 | CYP3A5 | -1.76765463351396 | 0.000618568266244194 |
| 282316 | POU5F1 | -0.649420475703755 | 0.0314182155829802 |
| 282326 | HTRA1 | -1.55395507726355 | 0.00537260192171739 |
| 282462 | MEA1 | -0.619496770029273 | 0.0419439872323889 |
| 282856 | PCK2 | -1.05621399894537 | 0.0247916557708796 |
| 338078 | STC1 | -1.91616924066044 | 0.0025267487664919 |
| 359715 | TRIM21 | -1.12639046938994 | 0.0452497316100024 |
| 404187 | CTSZ | -1.42737654826301 | 0.000456978252662449 |
| 504800 | APOBR | -0.696090859834712 | 0.0324134768210337 |
| 505438 | ARFGAP2 | -0.741347141191272 | 0.00300326766889341 |
| 505794 | KLHL41 | -8.24592470530832 | 0.0379430487631599 |
| 506560 | MYG1 | -0.906872305057483 | 0.0247916557708796 |
| 506759 | IFI16 | -2.79691857065936 | 0.0395497420919867 |
| 506790 | TXNIP | -2.38687844105742 | 0.0380014151765604 |
| 507473 | CNPPD1 | -2.01354092456356 | 0.0481300318783325 |
| 507839 | PRMT2 | -1.05232426274035 | 0.0454192355539663 |
| 507858 | SQOR | -2.78625663014948 | 0.000258101397837374 |
| 509255 | CDK5RAP3 | -1.09113095989406 | 0.003536309770115 |
| 513617 | ZNF622 | -0.701685837588947 | 0.0442698060999488 |
| 513621 | VAMP1 | -1.43023280592551 | 0.0247916557708796 |
| 514669 | WRAP73 | -1.10870202708404 | 0.0308825939194644 |
| 515472 | RECQL4 | -1.10163055622308 | 0.000949292308008263 |
| 516934 | RPUSD4 | -1.0560788044235 | 0.0247916557708796 |
| 518906 | GPR153 | -1.36680340202452 | 0.00642207964083551 |
| 522144 | C5AR2 | -8.21852212727869 | 0.0247916557708796 |
| 525414 | DNAH8 | -1.41989710151434 | 0.00581574424319183 |
| 527488 | SNTA1 | -1.8418663908841 | 0.0204876818383834 |
| 533145 | PHLDB3 | -1.22072139717579 | 0.0442698060999488 |
| 533356 | WDR18 | -0.731965270459376 | 0.0379430487631599 |
| 535109 | PACSIN3 | -0.922360317483158 | 0.00863845848214188 |
| 535992 | RAB11FIP5 | -1.95280950089108 | 0.0131776178972863 |
| 536153 | GPC6 | -2.56370135899589 | 0.00362238700101371 |
| 537379 | RBP1 | -1.44297678365868 | 0.032974345687821 |
| 538998 | FKBP2 | -0.75208732352853 | 0.0256475601051833 |
| 539976 | P3H1 | -1.38355010684836 | 0.0100355626037502 |
| 540229 | HSD3B7 | -1.36783463332751 | 0.0248041186692876 |
| 613571 | FUOM | -0.664135299290581 | 0.0283463964916156 |
| 614194 | CTU2 | -0.739240108071606 | 0.0390733116251348 |
| 614673 | NUPR1 | -1.49330625721758 | 1.42413816372017e-06 |
| 618333 | EFCAB9 | -10.0734557934217 | 1.42413816372017e-06 |
| 787106 | SERPINB12 | -8.49019693206925 | 0.0247916557708796 |
| 787811 | LGALS7 | -0.914158786630521 | 0.000618568266244194 |
| 788323 | OR2T29 | -8.41268400609522 | 0.0247916557708796 |
| 788925 | PRR5 | -0.77605363824037 | 0.0360981407957021 |
| 100125591 | IRF7 | -0.974055101557263 | 0.000527010519706765 |
| 100139548 | SKIV2L | -0.942399518869576 | 0.0325380956070901 |
| 100298130 | LGALS7B | -0.883290223525817 | 0.0411005113674903 |
| 100335527 | MEG3 | -0.890450389598758 | 0.0455514308707844 |
| 100337422 | SIM2 | -1.37748410337243 | 0.0452497316100024 |
| 107132821 | LOC107132821 | -8.41944370818012 | 0.0212974363721606 |
| 112445956 | LOC112445956 | -1.13682425795628 | 0.00287795461712212 |
| C5H12orf10 | C5H12orf10 | -0.905061743929376 | 0.0247916557708796 |
| ENSBTAG00000045898 | ENSBTAG00000045898 | -8.10448088825526 | 0.0472765497502034 |
| ENSBTAG00000050807 | ENSBTAG00000050807 | -6.09497804135329 | 0.0442698060999488 |
| ENSBTAG00000051369 | ENSBTAG00000051369 | -1.9686994410877 | 0.00181334586052221 |
| LOC100139548 | LOC100139548 | -0.972331549137166 | 0.0242718592012257 |
| LOC510351 | LOC510351 | -9.79928056120396 | 0.000258101397837374 |
| LOC788323 | LOC788323 | -8.41268400609522 | 0.0247916557708796 |

**Supplementary table 3.** DEGs upregulated in CON group compared with TOP group.

| ENTREZ_GENE_ID | Gene Symbol | Log2FoldChange | p-adjusted |
| --- | --- | --- | --- |
| 280832 | KIT | 0.85441331693115 | 0.0452497316100024 |
| 281170 | FRZB | 6.30665199518012 | 0.00583082281672741 |
| 281233 | IBSP | 8.63562132849618 | 0.0118196040904949 |
| 281760 | FABP5 | 1.23687700446521 | 0.015663990557753 |
| 281832 | HSP90AA1 | 0.557087807269018 | 0.0454192355539663 |
| 282023 | PTGS2 | 0.994842136031616 | 0.0223350608695638 |
| 282191 | COL4A1 | 0.81236668547163 | 0.0339872906832597 |
| 282301 | PDGFRA | 1.61085927900814 | 6.70305358372421e-06 |
| 282491 | BOLA-DMB | 9.57622907748399 | 7.77155795679665e-05 |
| 286800 | CACNG2 | 3.15739628748593 | 0.0113142993554135 |
| 407767 | HMGCS1 | 0.709940034026529 | 0.0247916557708796 |
| 504220 | SLC7A7 | 0.994285634903316 | 0.0311951768871797 |
| 504440 | EXOC1 | 0.940432626678191 | 0.0247916557708796 |
| 504657 | ARHGAP29 | 3.52109278891545 | 0.000446699087448344 |
| 506945 | BHLHE40 | 1.80839120361504 | 0.00287795461712212 |
| 509805 | LOC509805 | 8.41876269652628 | 0.0243552457888442 |
| 510366 | NIN | 1.13427042193159 | 0.0185700424499032 |
| 511167 | NRM | 1.24681703673015 | 0.0476303154723586 |
| 512512 | DNASE1L3 | 8.40602647474521 | 0.0223350608695638 |
| 512700 | FLT3 | 1.44222685313785 | 0.00018882961883593 |
| 513774 | AKAP12 | 0.859224650342642 | 0.00541595677826451 |
| 513856 | A2M | 1.39782027102093 | 0.00863845848214188 |
| 514255 | SLC20A1 | 0.863345915277968 | 0.0025267487664919 |
| 514261 | KIF5B | 0.870147485010398 | 0.00287795461712212 |
| 515333 | LOC515333 | 0.970249905637036 | 0.0247916557708796 |
| 526535 | SQLE | 0.909964727836089 | 0.0247916557708796 |
| 527740 | CBFA2T2 | 0.973429015287242 | 0.0325380956070901 |
| 529416 | FLRT3 | 1.6671595449296 | 0.0247916557708796 |
| 531682 | CAT | 1.09845098273371 | 0.0102657871154471 |
| 532600 | TMEM165 | 0.916333454769726 | 0.0247916557708796 |
| 532836 | AHCYL2 | 1.04310048995684 | 0.00287795461712212 |
| 533129 | ETV4 | 9.19872897121618 | 0.00181334586052221 |
| 534319 | NID1 | 1.63315606507379 | 0.000488168580846659 |
| 535194 | GPHN | 0.963705770609429 | 0.0247916557708796 |
| 536753 | CPN1 | 8.55326558111619 | 0.0137711073706202 |
| 538475 | PDZD4 | 9.21847309776031 | 0.000949292308008263 |
| 538951 | NANOG | 2.09977637297397 | 0.0247916557708796 |
| 539638 | FAM124A | 1.77297872288196 | 0.0102163721833037 |
| 540187 | EML4 | 1.17920286914634 | 0.0354534599619758 |
| 614007 | SH3BGRL | 1.11866298611983 | 0.0293835560448444 |
| 614456 | KRT6A | 3.47480312518008 | 0.0161988408726133 |
| 615304 | CPNE3 | 0.738088970969995 | 0.0457768637762308 |
| 615323 | GRP | 1.87663725698095 | 0.0152181006879066 |
| 782386 | NLRP14 | 9.09512348719037 | 0.0025267487664919 |
| 785309 | LOC785309 | 6.06300083336434 | 0.0025267487664919 |
| 100297935 | TMEM132D | 1.82036656314094 | 0.00942867238600327 |
| 101902345 | LOC101902345 | 8.54670923012809 | 0.0247916557708796 |
| 101903068 | LOC101903068 | 8.76315088118228 | 0.0047567544158746 |
| 101906311 | LOC101906311 | 3.51233432332272 | 0.049868331179072 |
| 101906545 | LOC101906545 | 7.44218888036709 | 0.0025267487664919 |
| 104970173 | LOC104970173 | 1.10471130709815 | 0.0113142993554135 |
| 104972133 | LOC104972133 | 8.46519927945911 | 0.0272056067302826 |
| 107131398 | LOC107131398 | 0.836216437736744 | 0.00362238700101371 |
| 107131637 | LOC107131637 | 8.16988899673858 | 0.0476303154723586 |
| 112443766 | LOC112443766 | 8.6391067415767 | 0.00863845848214188 |
| 112443788 | LOC112443788 | 8.09130576449352 | 0.00455387759179149 |
| 112445782 | LOC112445782 | 2.38887221408625 | 3.86927717694403e-07 |
| 112447077 | LOC112447077 | 7.88903892008004 | 0.0185700424499032 |
| 112449040 | LOC112449040 | 3.07439287541075 | 0.0399143414726624 |
| ENSBTAG00000012748 | ENSBTAG00000012748 | 8.41876269652628 | 0.0243552457888442 |
| ENSBTAG00000017978 | ENSBTAG00000017978 | 8.68880966629609 | 0.00583082281672741 |
| ENSBTAG00000040504 | ENSBTAG00000040504 | 6.0495390579515 | 0.00259471197539851 |
| ENSBTAG00000043567 | ENSBTAG00000043567 | 0.764340902589985 | 0.0260715716188983 |
| ENSBTAG00000043570 | ENSBTAG00000043570 | 0.71404946830421 | 0.0326498395466801 |
| ENSBTAG00000050318 | ENSBTAG00000050318 | 6.09135518904841 | 0.0450549648834077 |
| ENSBTAG00000053113 | ENSBTAG00000053113 | 8.37791306174914 | 0.0248041186692876 |
| ENSBTAG00000053900 | ENSBTAG00000053900 | 8.56457790657961 | 0.0113571064503454 |

**Supplementary table 4.** DEGs upregulated in ENC group compared with CON group.

| ENTREZ_GENE_ID | Gene Symbol | Log2FoldChange | p-adjusted |
| --- | --- | --- | --- |
| 280752 | CNP | -1.48024534258046 | 0.0267671453348643 |
| 280890 | PAM | -2.69223125957605 | 0.000587104390443897 |
| 281153 | FATE1 | -8.24964164905078 | 0.0165811067725598 |
| 281254 | INHA | -4.21617918808899 | 0.00616519788191649 |
| 281511 | SYT1 | -2.35865416872822 | 0.0407801696473033 |
| 281611 | AKAP4 | -8.33304545392209 | 0.0387201867269703 |
| 282013 | PSMB8 | -3.54668874557084 | 0.00127012596387676 |
| 282603 | PRSS2 | -3.23606491190031 | 0.0375135933009271 |
| 287026 | PLCB1 | -2.06132644306084 | 0.0324740625442519 |
| 338078 | STC1 | -1.88550579992378 | 0.0205263414967662 |
| 353510 | IFITM1 | -3.94569651558334 | 0.000701313817965669 |
| 404163 | ST3GAL4 | -2.14458415700886 | 0.0373561637338938 |
| 404187 | CTSZ | -1.48297480302979 | 0.0496893841636882 |
| 407107 | COL4A4 | -2.47389408689241 | 0.0205263414967662 |
| 444863 | GYPC | -3.74070078243492 | 0.00369302621387483 |
| 504407 | SPSB1 | -1.53886925962288 | 0.0205263414967662 |
| 505184 | SERPINB2 | -6.34279449465267 | 0.00222994781490459 |
| 505632 | MARCO | -3.19637565770419 | 0.0317650268914302 |
| 506415 | RSAD2 | -3.4437007312741 | 0.000701313817965669 |
| 506604 | ISG20 | -2.27139354002232 | 0.0126509397019394 |
| 506759 | IFI16 | -3.49041356251477 | 0.00175013053000843 |
| 507432 | MAP1LC3C | -3.71385617651416 | 0.0373601369986782 |
| 507858 | SQOR | -2.6749248080686 | 0.00889384205669585 |
| 508224 | SCG5 | -4.21457585231567 | 0.025003228900464 |
| 508268 | CLDN11 | -8.44705159185017 | 0.0170455340423395 |
| 508348 | IFI44 | -3.1955972881167 | 0.00347458223568008 |
| 509678 | IFIT3 | -6.73683415358899 | 3.25834255919767e-08 |
| 509823 | PALMD | -4.68062300444556 | 0.00229147455807307 |
| 510203 | CAVIN3 | -3.02324389359259 | 0.0205263414967662 |
| 510359 | TSC22D1 | -1.67986788070547 | 0.0440650857935916 |
| 510496 | PLAC9 | -2.8818232595547 | 0.000701313817965669 |
| 510658 | SLC2A6 | -4.3079439898393 | 0.00754885918462556 |
| 510773 | PID1 | -2.02066182679198 | 0.0212590282099714 |
| 511575 | GPX8 | -2.37830656620437 | 0.00347458223568008 |
| 511799 | P3H2 | -1.46511136355834 | 0.0496893841636882 |
| 512106 | MAP7D1 | -2.53161031243187 | 0.00429667176247899 |
| 512999 | MANSC1 | -3.72471209337387 | 0.00162840085976585 |
| 514394 | UCHL1 | -2.09220176286788 | 0.00369302621387483 |
| 514474 | TP53BP2 | -1.29199563159002 | 0.0262034058239338 |
| 514889 | IFNGR2 | -4.02080581780257 | 0.0202866413822475 |
| 515224 | HAPLN3 | -2.52456179395406 | 0.0440650857935916 |
| 515367 | FSTL3 | -1.84260251721589 | 0.0252000783864851 |
| 515593 | MAP1A | -2.01755025848115 | 0.00183024867340268 |
| 515731 | PLXDC2 | -1.63805128324987 | 0.0343953179310027 |
| 517545 | RGS6 | -2.82043633182319 | 0.0232807434293413 |
| 517768 | HS6ST2 | -3.17751207185448 | 0.00429667176247899 |
| 518368 | PARM1 | -1.89408585467031 | 0.00429667176247899 |
| 518845 | PRSS50 | -4.19795791948248 | 0.0266755501867102 |
| 519758 | ATP1B1 | -1.85804030571383 | 0.0223070282954232 |
| 522049 | CDC20B | -1.80347629720769 | 0.046363513592155 |
| 522392 | MXRA8 | -2.27620288015729 | 0.0205263414967662 |
| 526865 | PVR | -2.77621911212913 | 0.00283462082444857 |
| 528099 | RAB32 | -2.09026429933745 | 0.0407801696473033 |
| 530164 | SLC17A5 | -1.57141439302074 | 0.0496893841636882 |
| 532442 | RTP4 | -4.23621253004267 | 0.00369302621387483 |
| 534129 | CREB5 | -1.98454403695126 | 0.00582239253574006 |
| 534626 | GRIA3 | -1.81285293190426 | 0.0399899323158629 |
| 535439 | SMARCA1 | -2.76340326630871 | 0.0178071277165469 |
| 536607 | ACTN2 | -4.39956032612458 | 0.0267671453348643 |
| 537379 | RBP1 | -2.52275330227786 | 0.00183024867340268 |
| 538062 | EDIL3 | -3.29223195580721 | 0.00226688373582511 |
| 538437 | GEM | -3.63556438212541 | 0.010603280269374 |
| 538466 | TECTB | -8.22874523859037 | 0.0202866413822475 |
| 540318 | ZNF664 | -1.7423885573535 | 0.0170455340423395 |
| 613787 | PDGFC | -2.3540059179062 | 0.0196774650713736 |
| 614348 | RGCC | -3.99236869148379 | 0.0170455340423395 |
| 615813 | ITPKA | -2.41602267701928 | 0.0496893841636882 |
| 615929 | GGT7 | -8.88572791268194 | 0.00369302621387483 |
| 768028 | BEX2 | -1.29446366284484 | 0.0293655420011335 |
| 781681 | GLDN | -4.73341194502748 | 0.0359288262160204 |
| 781806 | FNDC4 | -4.44397350905126 | 0.00429667176247899 |
| 784747 | ZFPM2 | -2.88988627795985 | 0.0266755501867102 |
| 785475 | CDH11 | -2.54532949590765 | 0.0394178685396362 |
| 785924 | ROR2 | -2.98867130813389 | 0.000221595020301482 |
| 100125309 | TSC22D3 | -3.23505230827477 | 0.00889384205669585 |
| 100125760 | DPP7 | -2.86066514974358 | 0.00585042831481341 |
| 100140421 | GCKR | -2.47254720869902 | 0.046363513592155 |
| 100300400 | CLOCK | -1.64952843442841 | 0.0317650268914302 |
| 104968447 | HS3ST4 | -3.42776775382814 | 0.0267671453348643 |
| 112441657 | LOC112441657 | -7.64248491284883 | 0.0202866413822475 |
| 112442048 | LOC112442048 | -8.27875843277875 | 0.0196774650713736 |
| 112448058 | LOC112448058 | -5.86944403741009 | 0.00175013053000843 |
| ENSBTAG00000023666 | ENSBTAG00000023666 | -8.48185183849812 | 0.0122743818150494 |
| ENSBTAG00000051369 | ENSBTAG00000051369 | -1.9294103649497 | 0.0266755501867102 |
| ENSBTAG00000053138 | ENSBTAG00000053138 | -4.53876229138195 | 0.00582239253574006 |
| ENSBTAG00000054391 | ENSBTAG00000054391 | -3.06476597962763 | 0.0373601369986782 |

**Supplementary table 5.** DEGs upregulated in CON group compared with ENC group.

| ENTREZ_GENE_ID | Gene Symbol | Log2FoldChange | p-adjusted |
| --- | --- | --- | --- |
| 281246 | IL10 | 8.10921947640496 | 0.0399899323158629 |
| 282022 | PTGS1 | 4.64405974793318 | 0.0196774650713736 |
| 505671 | RFTN2 | 1.83186329425103 | 0.0387201867269703 |
| 506404 | CENPK | 1.25372267241712 | 0.0442470315738893 |
| 528021 | ANKRD13C | 8.4860558507193 | 0.0164913687494165 |
| 528647 | VSX2 | 8.06325191824682 | 0.0496893841636882 |
| 537150 | SLC14A2 | 8.74134604472184 | 0.0120885054006015 |
| 538731 | ALG8 | 1.38464074908324 | 0.0201847849441337 |
| 539420 | ELF5 | 6.15973746379416 | 0.0373601369986782 |
| 613358 | MT1E | 2.39003051721811 | 0.00120049834862865 |
| 784785 | ZNF473 | 2.12624056238199 | 0.0266755501867102 |
| 104975030 | LOC104975030 | 8.28692061237668 | 0.0373601369986782 |
| 112448786 | LOC112448786 | 7.89078503023794 | 0.0086706369404078 |
| ENSBTAG00000001442 | ENSBTAG00000001442 | 9.32711479622556 | 0.00114707017836248 |
| ENSBTAG00000017978 | ENSBTAG00000017978 | 8.69387861901925 | 0.00386490838973485 |
| ENSBTAG00000048434 | ENSBTAG00000048434 | 7.70894311236953 | 0.0170455340423395 |
| ENSBTAG00000053747 | ENSBTAG00000053747 | 1.3920242962898 | 0.0138858598428916 |

**Supplementary table 6.** DEGs upregulated in TOP group compared with ENC group.

| ENTREZ_GENE_ID | Gene Symbol | Log2FoldChange | p-adjusted |
| --- | --- | --- | --- |
| 515382 | SCRG1 | -8.88563194311224 | 0.0257743173024902 |
| 516043 | APOBEC1 | -9.51826074262124 | 0.000907580496405638 |
| 520023 | LOC520023 | -9.13187493145086 | 0.000868991889497393 |
| 520133 | ADAMTS16 | -6.6954494182941 | 0.0364380155610718 |
| 617830 | POU3F1 | -6.07150572446827 | 0.0275780255702947 |
| ENSBTAG00000003047 | ENSBTAG00000003047 | -9.63165099269118 | 0.000468084854840669 |
| ENSBTAG00000035530 | ENSBTAG00000035530 | -8.74586494211748 | 0.0275780255702947 |
| ENSBTAG00000048900 | ENSBTAG00000048900 | -8.82319847884434 | 0.0275780255702947 |
| LOC510351 | LOC510351 | -10.0286209547863 | 0.000868991889497393 |

**Supplementary table 7.** DEGs upregulated in ENC group compared with TOP group.

| ENTREZ_GENE_ID | Gene Symbol | Log2FoldChange | p-adjusted |
| --- | --- | --- | --- |
| 353510 | IFITM1 | 4.87127928705006 | 3.87785299463253e-05 |
| 506604 | ISG20 | 2.33124013482553 | 0.0275780255702947 |
| 507432 | MAP1LC3C | 2.36901220662002 | 0.0318208285093466 |
| 507859 | NCF4 | 8.82434791282612 | 0.0373133047828006 |
| 521580 | LOC521580 | 6.41737729813933 | 0.0275780255702947 |
| 531084 | PADI6 | 8.68464401919553 | 0.0368859112730354 |
| 531659 | C1QTNF3 | 3.1253713779423 | 0.0193993147175048 |
| 535376 | TGFBR2 | 1.90982364205933 | 0.0433531822649895 |
| 538691 | HS3ST1 | 3.9380982608576 | 0.0257743173024902 |
| 615254 | PHF24 | 4.85171209316065 | 0.0373133047828006 |
| 617314 | ZBTB37 | 9.75984922598326 | 0.000907580496405638 |
| 786372 | LOC786372 | 9.18509077518692 | 0.0109241447935517 |
| 100295249 | HOXC8 | 8.68562105184465 | 0.0364380155610718 |
| 101902345 | LOC101902345 | 8.85594387098515 | 0.0177007373513034 |
| 101906545 | LOC101906545 | 7.34710068889098 | 0.0249637555942031 |
| 112443766 | LOC112443766 | 8.69286296635011 | 0.0364380155610718 |
| 112446374 | LOC112446374 | 2.46257869978965 | 0.0318163699622503 |
| 112449112 | LOC112449112 | 8.09945734184364 | 0.0364380155610718 |
| ENSBTAG00000053070 | ENSBTAG00000053070 | 5.77887961544633 | 0.0499891835779205 |

**Supplementary table 8.** Intersection list of DEGs between group comparison.

| Gene Symbol | Log2FoldChange  CON_vs_ENC | Log2FoldChange  CON_vs_TOP | Log2FoldChange  ENC_vs_TOP |
| --- | --- | --- | --- |
| ENSBTAG00000017978 | 8.69387861901925 | 8.68880966629609 | - |
| IFI16 | -3.49041356251477 | -2.79691857065936 | - |
| SQOR | -2.6749248080686 | -2.78625663014948 | - |
| RBP1 | -2.52275330227786 | -1.44297678365868 | - |
| ENSBTAG00000051369 | -1.9294103649497 | -1.9686994410877 | - |
| STC1 | -1.88550579992378 | -1.91616924066044 | - |
| CTSZ | -1.48297480302979 | -1.42737654826301 | - |
| IFITM1 | -3.94569651558334 | - | 4.87127928705006 |
| MAP1LC3C | -3.71385617651416 | - | 2.36901220662002 |
| ISG20 | -2.27139354002232 | - | 2.33124013482553 |
| LOC510351 | - | -9.79928056120396 | -10.0286209547863 |
| LOC112443766 | - | 8.6391067415767 | 8.69286296635011 |
| LOC101902345 | - | 8.54670923012809 | 8.85594387098515 |
| LOC101906545 | - | 7.44218888036709 | 7.34710068889098 |
