# Supplementary material for: Transcriptomic effects of alginate hydrogel applied to the production of bovine embryos
Source: Heliyon. 2024 Dec 6;10(24):e40957. doi: 10.1016/j.heliyon.2024.e40957 (PMC11700250; doi:10.1016/j.heliyon.2024.e40957)
Supplement: Multimedia component 3 [file mmc3.docx]

***Results***

**Osmolarity test ENC and TOP groups**

As it is possible to see, cell culture the media changed in osmolarity as well as in color. We have tested the cell culture media osmolarity and it is affected by the TOP culture system. (Table 1 and 2). Apparently, changes caused by the ENC system are not drastically affecting the culture media. One of the possible reasons for that is the crosslink solution and the slow release of the ions during culture.

Table SM1: *In vitro* maturation media osmolarity.

|  | mOsm/Kg H2O | Average  mOsm/Kg H2O |
| --- | --- | --- |
| Control_IVM | 292 | 295 |
| Control_IVM | 300 |  |
| Control_IVM | 293 |  |
| TOP_IVM | 244 | 249 |
| TOP_IVM | 244 |  |
| TOP_IVM | 258 |  |
| ENC_IVM | 302 | 310 |
| ENC_IVM | 305 |  |
| ENC_IVM | 324 |  |

| Ttest | p-value |
| --- | --- |
| ControlVsTOP_IVM | 0.001 |
| ControlVsENC_IVM | 0.105 |

Table SM2: *In vitro* culture media (SOF)

|  | mOsm/Kg H2O | Average  mOsm/Kg H2O |
| --- | --- | --- |
| Control_SOF | 283 | 304 |
| Control_SOF | 310 |  |
| Control_SOF | 318 |  |
| TOP_SOF | 273 | 270 |
| TOP_SOF | 270 |  |
| TOP_SOF | 266 |  |
| ENC_SOF | 312 | 317 |
| ENC_SOF | 317 |  |
| ENC_SOF | 322 |  |

| Ttest | p-value |
| --- | --- |
| ControlVsTOP_SOF | 0.03 |
| ControlVsENC_SOF | 0.29 |

In addition to that we have added images from the media after IVM and IVC in the alginate ENC and TOP as well as in the control group. Images:


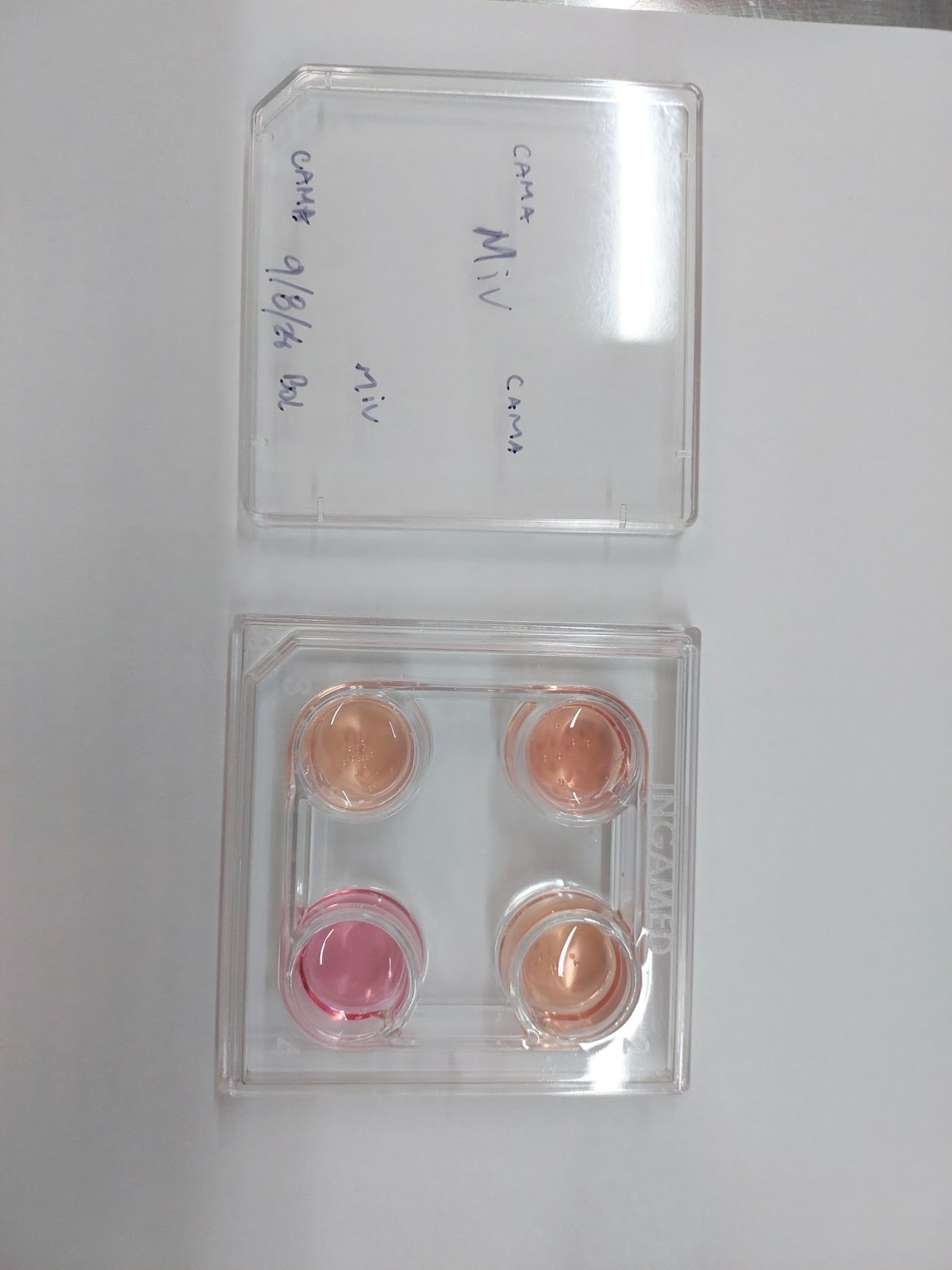


4

3

2

1

Figure SM1: On top and control group. *In vitro* maturation media (Wells 1, 2, and 3 are on top group and well 4 is the control media).


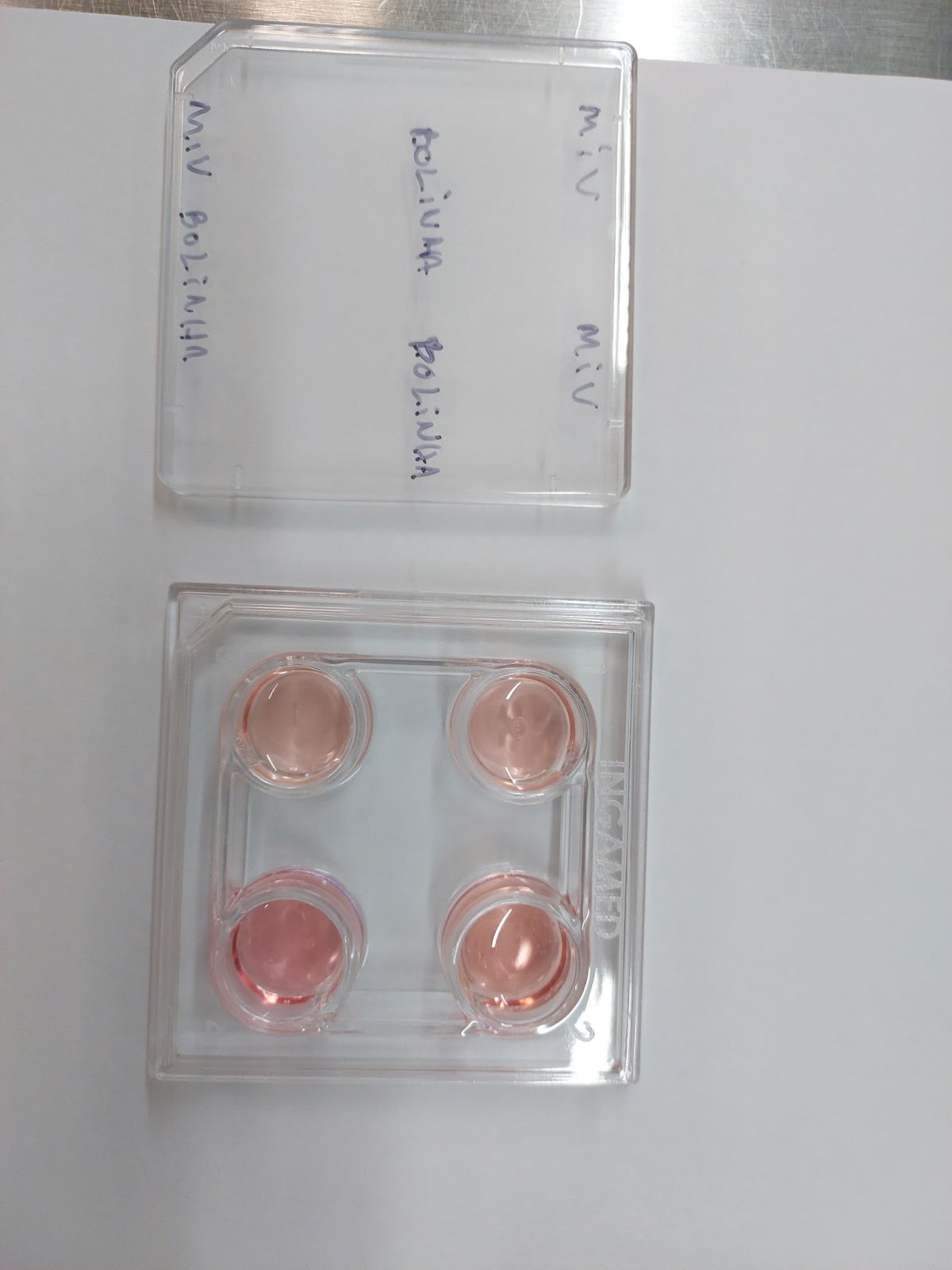


1

2

3

4

Figure SM2: Encapsulated and control group. *In vitro* maturation media (Wells 1, 2, and 4 are encapsulated group and well 3 is the control media).


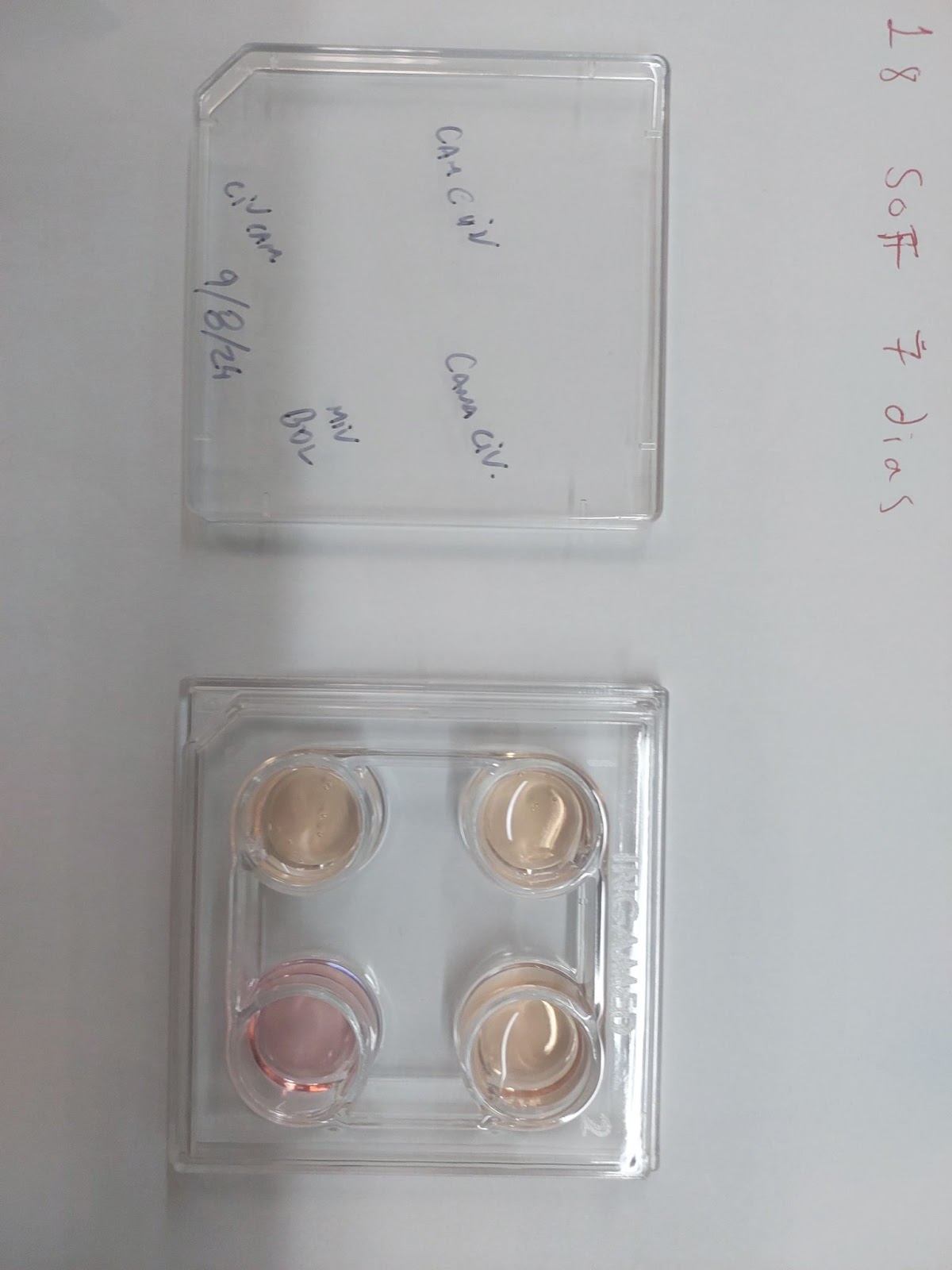


4

3

2

1

Figure SM3: On top and control group. *In vitro* culture media (Wells 1, 2, and 3 are on top group and well 4 is the control media).


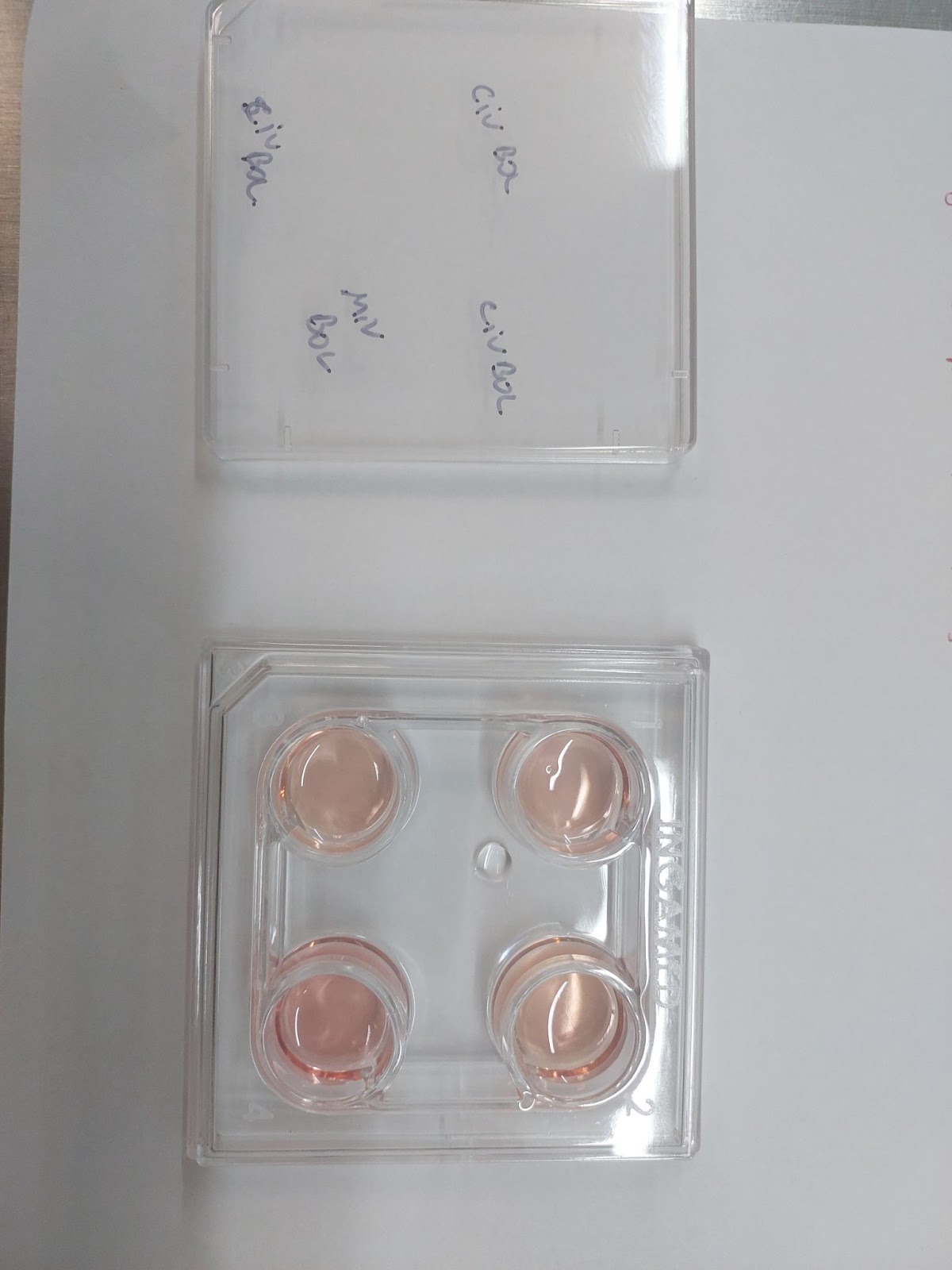


Figure SM4: Encapsulated and control group. *In vitro* culture media (Wells 1, 2, and 3 are encapsulated group and well 4 is the control media).
